# Dried Plasma for Major Trauma: Past, Present, and Future

**DOI:** 10.3390/life14050619

**Published:** 2024-05-10

**Authors:** Henry T. Peng, Kanwal Singh, Shawn G. Rhind, Luis da Luz, Andrew Beckett

**Affiliations:** 1 Defence Research and Development Canada, Toronto Research Centre, Toronto, ON M3K 2C9, Canada; kanwal.singh@drdc-rddc.gc.ca (K.S.); shawn.rhind@drdc-rddc.gc.ca (S.G.R.); 2 Sunnybrook Health Sciences Centre, University of Toronto, Toronto, ON M4N 3M5, Canada; luis.daluz@sunnybrook.ca; 3 St. Michael’s Hospital, University of Toronto, Toronto, ON M5B 1W8, Canada; andrew.beckett@unityhealth.to; 4 Royal Canadian Medical Services, Ottawa, ON K1A 0K2, Canada

**Keywords:** blood transfusion, coagulation, dried plasma, freeze-dried plasma, hemorrhage, major trauma

## Abstract

Uncontrollable bleeding is recognized as the leading cause of preventable death among trauma patients. Early transfusion of blood products, especially plasma replacing crystalloid and colloid solutions, has been shown to increase survival of severely injured patients. However, the requirements for cold storage and thawing processes prior to transfusion present significant logistical challenges in prehospital and remote areas, resulting in a considerable delay in receiving thawed or liquid plasma, even in hospitals. In contrast, freeze- or spray-dried plasma, which can be massively produced, stockpiled, and stored at room temperature, is easily carried and can be reconstituted for transfusion in minutes, provides a promising alternative. Drawn from history, this paper provides a review of different forms of dried plasma with a focus on in vitro characterization of hemostatic properties, to assess the effects of the drying process, storage conditions in dry form and after reconstitution, their distinct safety and/or efficacy profiles currently in different phases of development, and to discuss the current expectations of these products in the context of recent preclinical and clinical trials. Future research directions are presented as well.

## 1. Introduction

Hemorrhage accounts for about 90% of preventable death in combat trauma [1,2,3], and is the secondary cause of death in civilian trauma with up to 40% mortality [4,5]. Coagulopathic bleeding is frequently present early after major trauma (one in four patients at admission) with three to five times higher mortality [6,7]. In addition, a significant number of deaths due to hemorrhage occur in the first several hours after injury in both military and civilian trauma [8], with 33–56% of prehospital deaths [9] and approximately 85% of deaths within 6 h after hospital arrival [10]. Early diagnosis of trauma-induced coagulopathy (TIC) and optimal coagulation management of the critically injured patients dictate emergent interventions to immediately stop bleeding with hemostatic agents and simultaneous transfusion of blood products, including whole blood (WB), red blood cells (RBC), fresh frozen plasma (FFP), freeze-dried plasma (FDP), clotting factor concentrates, cryoprecipitate, and platelets [11,12,13,14]. As a result, there has been an increased interest in hemostatic resuscitation in prehospital settings, the so-called ‘remote hemostatic resuscitation’ [15,16,17], which imposes logistical challenges for a blood product that can be easily stored, transported, and used in a field environment.

FFP is the preponderance of plasma used to restore deficient coagulation factors, but is not ideal for use in far-forward combat environments due to logistical reasons. When stored at −20 °C, FFP has an expected shelf-life of two years [18]. FFP must be thawed and warmed to prevent hypothermia prior to transfusion. The thawing process requires a 30–37 °C agitated water bath or a warming device and takes approximately 30 min. After thawing, FFP must be transfused immediately or refrigerated and used within five days [19]. There is also a significant loss due to bag breakage during transport and thawing [20]. This presents significant logistical challenges, resulting in a considerable delay in patients receiving plasma. Certain steps taken by major trauma centers to remedy that have been successful, including keeping thawed plasma ready in the emergency department at all times, as well as carrying thawed plasma or never-frozen plasma in the prehospital setting. These measures are costly and require a large-scale operation [21]. Smaller hospitals will not be able to accommodate such measures. In most countries, like Canada, only FFP is available.

Alternatively, FDP remains physiologically relevant as it retains the benefits of its source plasma, thereby preventing hemodilution and acidosis, as well as possessing hemostatic properties due to the inclusion of all the coagulation factors [22,23]. Both FFP and FDP can correct the endotheliopathy of trauma; however, FDP can provide technical and logistical advantages over FFP and liquid plasma for remote hemostatic resuscitation in prehospital settings and massive casualty events given its long storage stability, easy carry on, and fast reconstitution and administration [22,23]. In addition, dried blood products may enable cost savings given the potential to reduce blood product wastage by unused pre-thawed materials in rescue vehicles, helicopter services, and in early in-hospital trauma units [21]. Utilizing FDP allows for a rapid and high-ratio transfusion of plasma to RBC in severe trauma patients, leading to improved clinical outcomes [24,25].

Strategically, the COVID-19 pandemic and possible large conflicts or multi-domain operations in the foreseeable future highlight the need to stockpile blood products with long shelf lives (e.g., dried plasma), to provide sufficient medical care in an emergency and civilian disaster [26] and for massive combat casualties [27]. Various measures were adopted to address the need for blood supply due to the lockdown of blood donation centers and reduced medical resources during the COVID-19 pandemic [28].

Future multi-domain combat operations will increase the likelihood of massive casualties and prolonged field care, and reduced air superiority will further prohibit immediate evacuation and promote the need to medically support casualties in far-forward austere environments [29]. Moreover, remote environments impose further challenges for liquid blood products that must be refrigerated, properly stored, and have short storage lives. Given the benefits of early plasma resuscitation and the current state of technology, dried plasma is the most proximate and feasible solution to provide blood products when and where they are needed [30].

The logistic and strategic advantages are not only relevant for what may be called conventional warfare but are also important for other types of military operations, such as civilian mass-casualty events, counterinsurgency, and humanitarian assistance, depending on geography and the tactical situation where infrastructure may be compromised and local supplies overwhelmed [31]. There are also remote civilian hospitals and medical facilities around the world that are challenged by plasma availability and long prehospital transport times or limited by a lack of laboratory and storage facilities.

A number of reviews on FDP have been published [22,32,33,34], including ours, which focused on the studies of FDP for hemostatic resuscitation in both hemorrhagic animal models and trauma bleeding patients [32]. The current paper provides an overview of past and present developments in dried plasma with a focus on in vitro studies of hemostatic properties before and after drying, and stabilities during dry storage and after reconstitution. Furthermore, this paper provides a comparison of various dried plasma and delves into the future of dried plasma technology.

## 2. History of FDP

FDP has a long history of civil and military use that dates back to the 1940s [35,36]. At the time, serum was preferred compared to plasma, as the removal of clotting factors extended the shelf life of serum. In plasma, the fibrin and use of anticoagulant made drying very slow [37] and caused clot formation both before drying and after reconstitution. Serum was intended for volume expansion; consequently, the importance of the presence of clotting factors when treating massive hemorrhage might not have been fully appreciated [38,39].

The initial work involves the development of freeze-drying techniques [37,40] and infusion in experimental animal models in the 1930s [41,42], and in humans in the 1940s [43,44], leading to large-scale production with millions of units distributed to the Allied forces worldwide [45]. These dried units of plasma or serum were utilized as a primary mode of resuscitating combat casualties in World War II [33]. However, hepatitis transmission, with the use of pooled plasma or serum, led to the cessation of large-scale production by the end of the war [46] and the eventual abandonment of dried pooled plasma in the US by 1968 [39,47]. The French Military Blood Institute started its FDP production in 1950 and provided nearly 40,000 units of FDP to the French military during the Indochina War, but discontinued the production in 1985, due to risk of human immunodeficiency virus (HIV) infection from the pooled plasma used in FDP [48].

With the introduction of a robust hemovigilance program, there has been a significant improvement in donor screening, testing procedures, pathogen reduction, and freeze-drying technologies, since early 1990s [45,48], led by the French Blood Bank [48] and the German Red Cross [49], respectively. These advances have renewed interest in FDP as a modern, sterile, safe, and readily reconstituted blood component for hemostatic resuscitation, especially in prehospital and remote or austere environments. Discoveries of new human viruses, the implementation of new technologies for virus detection to exclude infected individuals, and the constitution of pools of repeat donors over the past two decades have significantly increased the safety of blood products and reduced the risk of transfusion-associated viral infections [50,51].

The French modern FDP production started in 1994, utilizing small donor pools (under 11 donors) and amotosalen with UV light processing for pathogen reduction [48]. Pooling based on blood type selection allows the dilution and neutralization of natural anti-A and anti-B hemagglutinins, making French FDP (FLyP) a universal donor product compatible with any recipient blood type. Since 2003, FLyP has also been leukoreduced. Starting in 2010, FLyP excludes plasma from women with human leukocyte antigen (HLA) antibodies. This step was introduced to mitigate the risk of transfusion-related acute lung injury (TRALI), a potentially fatal complication affecting the respiratory system [52]. The presence of HLA antibodies in females has been linked to immune responses during pregnancy [52]. Additionally, in 2010 the product further incorporated a pathogen reduction step to enhance safety. That was at the same time that FLyP started undergoing amotosalen and UV light processing as a pathogen DNA/RNA inactivation method. This process was chosen over solvent/detergent (S/D) treatment due to better preservation of clotting factors. The French hemovigilance program has been monitoring FLyP since 1994, and so far, no reactions or infectious complications have been reported for military and limited civilian use [48]. In July 2018, FLyP was approved by the US Food and Drug Administration (FDA) for limited emergency authorization use by the US military [53,54].

During the same time, the German Red Cross started processing pooled plasma with a S/D treatment as a method of pathogen inactivation [55]. This continued through the early 2000s when possible prion disease transmission in rare cases by blood components was recognized [56], which could not be inactivated by standard S/D treatment [49]. This led to a switch from pooled to single-donor plasma stored frozen for at least four months until the donor returns for retesting for HIV, hepatitis C virus, hepatitis B virus, hepatitis A virus, and parvovirus B19 [49]. The current German FDP (LyoPlas) began in 2007. It is ABO-specific and used in both military and civilian settings with excellent safety profiles.

**Figure 1:**
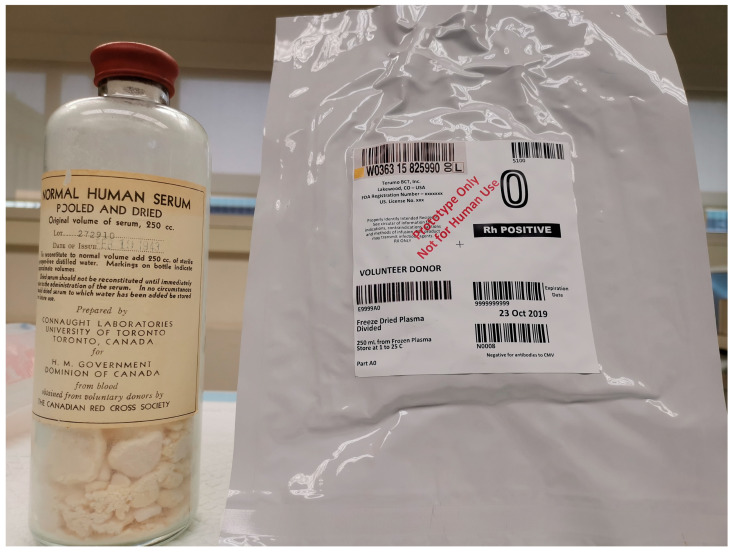
An original bottle of Canadian freeze-dried serum produced in 1943 (**left**) and a package of Canadian freeze-dried plasma (CFDP) produced in 2019 (**right**).

Canada has a long history of leading the development of freeze-dried blood products [39]. All development and production of freeze-dried serum in Canada during World War II involved University of Toronto’s Connaught Laboratories, led by Dr. Charles H. Best, which produced 400,000 bottles of dried serum for distribution to Canadian and Allied forces (Figure 1). Production stopped after the war due to the aforementioned risk of pathogen transmission. Interestingly, our recent analysis of an 80-year-old Canadian freeze-dried serum from World War II identified 71 proteins, the most prominent being albumin, as well as active antithrombin, plasminogen, protein C, and protein S; it was also shown to be positive for hepatitis B by serological testing [57].

**Figure 2:**
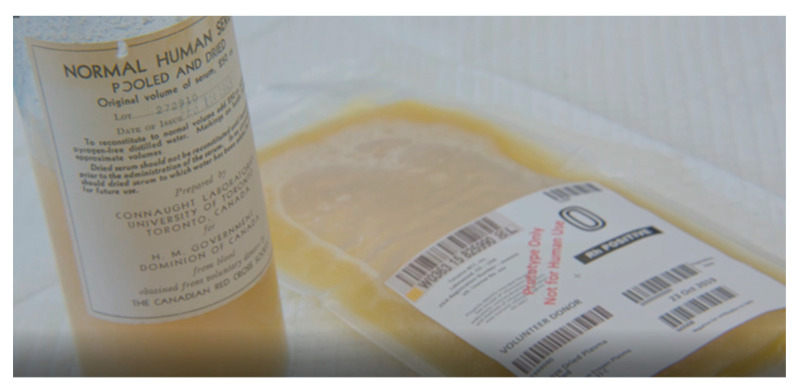
The original bottle of Canadian freeze-dried serum produced in 1943 (**left**) and a package of Canadian freeze-dried plasma produced in 2019 (**right**) after reconstitution with 250 mL of sterile water.

In the past few years, the Canadian Armed Forces, along with Canadian Blood Services and Defence Research and Development Canada, has been developing FDP from Canadian-sourced plasma using a new freeze-drying technology (Figure 1). The prototype kit consists of one unit of FDP and 250 mL of sterile water, each stored in a plastic bag that can be connected directly to transfer the water for rapid reconstitution and administration (Figure 2). We conducted a series of in vitro tests and demonstrated the equivalent clotting capacity of Canadian FDP (CFDP) to its source plasma [58,59], supporting its further development as a modern, sterile, readily reconstituted blood component in durable plastic bags for prehospital use in remote or austere environments [60]. Through this innovation, Canada can continue to contribute to advances in transfusion medicine, such as the development and deployment of FDP, as was the case during World War II.

## 3. Current Status of Dried Plasma

Both commercial and in-house dried plasma have been produced using two drying processes: freeze-drying under vacuum (lyophilization) and spray-drying. The former involves freezing plasma under a vacuum for several days and reducing water content to approximately 1–2% by sublimation [49]. The latter is conducted by the atomization of liquid plasma to droplets and brief exposure to hot (up to 150 °C) gas in a drying chamber, followed by rapid evaporative cooling [61].

Various types of plasma have been used to produce dried plasma: FFP is plasma frozen at −20 °C within 8 h of collection, PF-24 is plasma frozen at 24 h of collection, liquid plasma (LP) is never-frozen plasma but rather stored at 2 °C to 6 °C for up to 28 days, and S/D-treated plasma is made from pools of approximately 1000 FFP units [62]. Plasma can be obtained from a single donor or pooled from multiple donors by separating plasma from WB units or by apheresis collection, and then it is leukocyte reduced after collection. Pooled plasma mitigates the potential donor variability observed in single-donor plasma products (FFP and LP) [63]. The resultant dried plasma mimics the critical characteristics of their respective plasma source, in terms of clotting profiles, and effects on plasma oncotic pressure [23,64], as well as endothelium protection [65].

Dried plasma can be reconstituted to its original volume or a concentrated form for use by adding the supplied reconstitution fluid, typically sterile water, into the bottle or bag containing the plasma. The mixture is gently swirled and ready for infusion through a standard transfusion set and filter within 5 to 10 min. The standard sizes are 50 and 200 mL. The current cost of FDP is approximately two to three times more than frozen plasma [22] due to additional drying steps. It was estimated the theoretical cost of FDP for in-hospital use could raise expenses [66]; however, FDP may provide a solution for remote in-hospitals, where blood product accessibility is limited.

As summarized in Table 1, three different products of dried plasma are commercially available [45,67]: French FDP (FLyP), manufactured by the French Blood Bank from approximately 10 carefully screened and monitored donors [48,68,69,70]; German FDP (LyoPlas), manufactured by the German Red Cross from a single donor quarantined for at least four months and negatively tested for HIV and hepatitis B and C virus [23,49,71]; Bioplasma FDP, produced by the National Bioproducts Institute of South Africa from hundreds of donors (up to 1500) [72,73].

These products are all freeze-dried and produced by central production facility [22]. Each of these products meets quality standards and has been used with clinical success for the same indications for use as their source plasma or other forms of plasma or pathogen-reduced plasma. The key shortcoming of these approved products, as well as some in development for the US market, lies with their availability, which is due to a dependence on centralized manufacturing [74].

These products differ in various aspects:

- Starting plasma: LyoPlas—single-donor FFP; LyoPlas is ABO-specific and has unit-to-unit variability similar to single-donor FFP. FLyP and Bioplasma—mini- and large-pooled plasma; pooled, pathogen-reduced FDP (FLyP, Bioplasma FDP) are ABO-universal but have improved unit-to-unit consistency.
- Pathogen reduction: LyoPlas—not valid. FLyP—amotosalen with UV light. Bioplasma FDP—S/D treatment.
- Freeze-drying process and water content: LyoPlas—maintains a water content below 1% through its freeze-drying process.
- pH after rehydration: FLyP—achieves a pH of 8.
- Shelf life: LyoPlas—15 months under storage conditions of 2–25 °C. FLyP—two years under similar storage conditions.
- Other: differences in the type of additive(s) may affect stability, functional profile and biological composition have also been mentioned.

Retrospective studies have shown the feasibility, safety, logistic benefits, reduction in RBC transfusion and time to transfusion, and the positive effects on coagulation profiles when FLyP or LyoPlas were used for combat casualties in prehospital and austere environments [22,23,48,54,68,75,76,77,78]. However, randomized clinical trials (RCTs) comparing the transfusion of FLyP or LyoPlas with standard care in trauma resuscitation only found faster delivery of FDP (FLyP) compared to that of FFP (median time 14 versus 77 min), leading to a higher fibrinogen concentration 45 min after randomization compared with FFP, and a greater improvement in prothrombin time (PT) ratio, factors II, and V levels [79], but no differences in mortality [79,80,81]. However, a pilot RCT of 19 patients with suspected hemorrhage and hypovolemia showed that prehospital transfusion of two units of LyoPlas with RBC during helicopter transit to a trauma center was likely to reduce mortality at 24 h and hospital discharge with no serious adverse events compared to only RBC transfusion [82]. It was noted that time from randomization to hospital arrival in the UK was 36 min, compared to 92 min in the Australia where the study was conducted. Additionally, TXA was not administered in the prehospital setting, which could provide a deeper understanding into the role of blood products in the absence of antifibrinolytics.

Since 1996 in South Africa, Bioplasma FDP has been successfully transfused in all types of patients with a strong record of safety [72,73]. However, limited data are available in the peer-review literature, with one study showing the immediate efficacy and safety of Bioplasma FDP in cardiopulmonary bypass patients [83]. There is insufficient evidence of a 24 h mortality difference among trauma patients at risk for hemorrhage who received Bioplasma FDP alone, any other blood products alone, or any combinations [84].

We have conducted a systematic review and meta-analysis of FDP for major trauma in 12 human trials and 15 animal studies up to 31 March 2020 and confirmed that the use of FDP was feasible and safe even in austere and prehospital settings [32]. Human data showed no difference in mortality and transfusion of allogeneic blood products in patients receiving FDP compared with frozen plasma. Furthermore, laboratory measures of coagulopathy (INR, PT, and/or TEG/rotational thromboelastometry—ROTEM) in human and animal studies reported similar improvements in coagulation parameters when FDP was transfused compared with FP, suggesting retained coagulation profiles in preparation and after reconstitution. Additionally, data from animal trauma studies reported no difference in coagulation factor and anti-inflammatory profiles between FP and FDP.

Critical review of current high-level, randomized, controlled evidence of FDP for prehospital transfusion in trauma supported its feasibility and safety, and recommended further research to determine under what conditions/scenarios FDP might provide clinical benefits to a subset of trauma patients [85]. The lack of clinical superiority of FDP over saline could be explained by the divergent outcomes of two RCTs for prehospital thawed plasma transfusion [86,87]. Collectively, these studies suggest that blood products, including dried plasma, are effective in managing hemorrhagic shock when administered as close as possible to the time of injury, thereby improving short-term survival within a 6 h window [88]. Consequently, the efficacy of blood products beyond 6 h may not yield added benefits.

**Figure 3:**
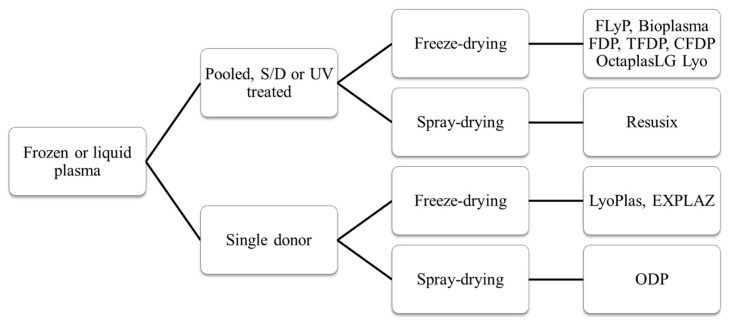
Main productive steps of various dried plasma products.

In addition, dried plasma has recently been produced using a spray-drying method and a decentralized, blood center-based manufacturing model [74]. As summarized in Table 1 and Figure 3, there are several freeze- and spray-dried plasma products under development [45,89,90,91,92], such as the following:

- EZPLAZ, Teleflex—a single-donor FDP [93,94,95].
- TFDP, Terumo BCT—single-donor or pooled FDP [58,59,91,94].
- OctaplasLG Lyo, Octopharma—pooled and S/D pathogen-reduced FDP [92,96].
- Resusix, Entegrion—S/D pathogen-reduced and pooled (100–1500 units) spray-dried plasma [45,97].
- Frontline On-Demand Plasma (ODP), Velico Medical—single-donor spray-dried plasma [61,98,99].

EZPLAZ, OctaplasLG Lyo, and Resusix are designed to be produced under centralized models as FLyP, LyoPlas and Bioplasma FDP. Terumo BCT FDP and Velico Medical ODP are manufactured through decentralized production by blood centers. EZPLAZ, Terumo BCT FDP, and ODP are expected to be ABO type-specific, while Octaplas Lyo and Resusix are projected to be universal. Initially, a single-donor, tested, FDP was developed by HemCon Medical Technologies, Inc. (Portland, OR, USA) [100] and was successful in a safety clinical trial of ascending doses in normal healthy subjects [101,102]. The development of HemCon ended in 2013, for reasons pertinent to business operations.

These new-generation FDP products have incorporated changes in formulation and packaging. These products can be produced using centralized manufacturing capabilities and distributed manufacturing systems at blood collection centers. Another significant alteration for the new-generation dried plasma is the package, which has been switched from glass bottles to plastic IV bags for rapid reconstitution, allowing for immediate infusion [93]. The package differences between plastic bag and glass bottle should not be underestimated, given that they could affect their performance in the field. Bag-formulated FDPs are more durable and suitable for remote austere settings as there is no breakage; they are easier to mix homogeneously in the field and can be administered more rapidly than glass-bottled FDPs, by massaging and squeezing the bag during reconstitution and transfusion. However, they tend to be less alkaline in pH than glass-bottled FDPs. Reconstituted bottled FLyP has a pH of 8 [69], while pH values of 7.80 ± 0.14 [91], 6.9 ± 0.2 [93], and 7.53 ± 0.06 [94] have been reported for the newer FDPs following the reconstitution of plasma containing a citrate-phosphate-dextrose (CPD) anticoagulant. A pH adjustment step was implemented by using citric acid, phosphoric acid, and low pressure, to compensate for a potential pH increase during the freeze-drying process [92]. Maintaining physiological pH may be important in mediating a beneficial effect of plasma transfusion in trauma treatment, although it seems likely that such differences would dissipate on infusion. Efforts should be made to limit the amount of citrate infused into a patient in hemorrhagic shock and thus the citrate load should be reduced, while simultaneously addressing the induced hypocalcemia [103].

Stabilizing additives may be used in the manufacturing process to protect plasma proteins from denaturation during both the freezing and the dehydration steps of the freeze-drying process. For example, glycine is added at 5 mg/mL final concentration to stabilize plasma proteins during freezing in the OctaplasLG manufacturing process [92]. Liquid plasma was added with a glycine–hydrochloric acid solution for the production of spray-dried plasma [98].

Pooled plasma standardizes factor activities with lower batch-to-batch variability compared to wide donor variability in single-donor plasma products. However, pooled plasma may have higher infectious risk than single-donor plasma unless a reliable pathogen inactivation process is used.

The challenges facing the aforementioned dried plasma products under development include regulatory issues, logistical issues, product issues, implementation, and reimbursement issues [104].

**Table 1:** Characteristics of modern dried plasma commercially available and under development.

Name	Plasma Source	Production Process and Reconstitution	Properties	Current Status	References
French FDP (FLyP)	Pooled apheresis plasma (mixed of A, B, and AB blood group) from <11 donors screened. Plasma quarantine and donor retesting, and a robust hemovigilance program via hemovigiliance program, leukocyte reduced, no HLA antibody women; ultraviolet light process (amotosalen) for pathogen inactivation.	Quickly frozen in a glass bottle before being freeze-dried in a freeze dryer for 4 days with no need for additional compound. Reconstituted in 200 mL of water with a transfer kit within 3–6 min, allowing for immediate transfusion with a transfusion kit with vented air intake.	Normal clotting factor levels, ABO-universal, shelf-life of 2 years at RT, the fibrinogen and clotting factor levels of FLyP are equivalent to FFP	Use by French Military since 1994, US military since 2018; licensed by ANSM (Agence nationale de sécurité du médicament et des produits de santé) for civilian use in France since 2012. No reactions or infectious complications have been reported out of more than 1100 units transfused since 1994.	Cuenca et al., 2020 [54]; Curry et al., 2019 [67]; Daban et al., 2010 [70]; Martinaud et al., 2011 [68] and 2012 [69]; Sailliol et al., 2013 [48]
German FDP (LyoPlas)	Single-donor plasma. Screened via hemovigiliance program, quarantine-stored for at least 4 months, leukocyte reduced. No HLA antibody in women.	One unit of plasma (200 mL) transferred and filtered to a glass bottle via steam-sterilized “bottle-in-bag” system is frozen below −30 °C in a separate step, followed by lyophilization in specially designed freeze dryers by a stepwise increase in the temperature from −45 °C to 15 °C. Reconstituted in 200 mL water within 10 min or less depending on the individual plasma composition (proteins and lipids) and the temperature of the water bag.	Normal clotting factor levels, ABO type specific, shelf life of 24 months at 2–8 °C, most factors remained stable, with only 10% reduction in FV, VIII and vWF, while storage at RT led to 54% decrease in fibrinogen levels and vWF activity, recommended shelf life of 15 months.	Use in general population in German, Israeli, and UK militaries, as well as in civilian population. Used by Norwegian helicopter EMS. The rates and types of transfusion-related complications were similar for FFP and LyoPlas. No viral transmission has been reported since its inception in 2007	Curry et al., 2019 [67]; Gokhale et al., 2016 [71]; Glassberg et al., 2013 [23]; Bux et al., 2013 [49]
Bioplasma FDP (National Bioproducts Institute, Pinetown, South Africa)	Pooled plasma from up to 1500 donors. Screened and comprehensive tested via hemovigilance program, solvent detergent treatment for pathogen inactivation.	Freeze-dried in 50 mL and 200 mL glass bottles. Reconstituted with either 50 or 200 mL of water in a glass bottle in <10 min (median time of 1.5 min).	Clotting factor levels ≥040 IU/mL, ABO-universal, shelf life of 2 years when stored below 25 °C. Contraindicated in severe protein S deficiency, no increase in adverse events compared to FFP.	Use in general population and all types of patients in South Africa and surrounding countries since 1996 with a high degree of safety.	Chapanduka et al., 2002 [83]; Curry et al., 2019 [67]; Solheim et al., 2008 [72]; Wise et al., 2020 [73]
Next-generation single-donor FDP (RePlas/EZPLAZ) manufactured by Vascular Solution LLC (acquired by Teleflex Inc., Wayne, PA, USA)	Single-donor apheresis-derived FFPs (ACD-A anticoagulant) and whole blood-derived FFPs (CPD anticoagulant).	Magnum shelf freeze dryer (Millrock Technology) in plastic IV bag containers. Reconstituted with approximately 260–270 mL (250 mL bag with overage) of sterile water for injection through fluid transfer set.	The average time of reconstitution was 67 s (range: 43–106); FDP coagulation factors, clotting times, and product quality (pH, total protein, and osmolality) post-lyophilization were preserved, ABO type specific.	Clinical dose escalation safety trial in healthy volunteer subjects.	Cancelas et al., 2022 [93]; Pusateri et al., 2019 [90]; Pusateri et al., 2022 [22]; Teleflex 2021 [95]
Terumo BCT FDP (TFDP)	Fresh frozen plasma (FFP) units were thawed, sampled, pooled in groups of 10 units or single donor	Using Terumo’s freeze-drying system in a durable, light-weight plastic package suitable for field use. Reconstituted within 3 min with 250 mL water for injection to provide a 1:1 reconstitution ratio back to the original plasma volume.	Coagulation factor activities, which were within clinical ranges, ABO type specific, stored for up to 2 years at room temperature and 4 °C.	Not approved for human use.	Flaumenhaft et al., 2021 [91]; Pusateri et al., 2019 [90]
Canadian FDP (CFDP)	10 units of Canadian-sourced FFP were pooled using a kit provided by the manufacturer (Terumo BCT).	Using Terumo’s freeze-drying system in a durable, light-weight plastic package suitable for field use. Reconstituted with 250 mL sterile water in less than 5 min	Equivalent to initial frozen plasma with respect to global hemostasis. Changes in coagulation and fibrinolysis factors were <15%; ABO type specific.	Not approved for human use.	Peng et al., 2021 [58]; Sheffield et al., 2022 [94]
Single-donor FDP (HemCon)	Derived from licensed FFP from nonremunerated single-donor volunteer donors. Transferred into a container consisting of spike ports, semi-permeable GORE^®^ membrane, heat transfer film and a novel closure system, lyophilized using a unique cycle that results in a stable, dry cake in a closed container system	Licensed FFP is aseptically placed into single-unit lyophilization containers and frozen under vacuum for 4 to 6 days in a commercial-scale lyophilizer and stored in a ruggedized administration container suitable for the field. Reconstituted with 200–250 mL of pH-adjusted sterile water in 2 min	Factors within normal range, with a loss of less than 15% activity for all measured factors, compared to FFP, the retention for FVIIa (−14%), protein S (12%) and F1+2 (28%) appeared to have larger changes.	Successful phase I clinical trial with no serious adverse events or safety concerns. Further development discontinued due to business reasons	Cancelas et al., 2011 [101]; Winata et al., 2011 [100]; Pusateri et al., 2016 [45]; HemCon 2012 [102]
Pooled S/D-treated FDP (OctaplasLG Lyo)	S/D-treated coagulation active plasma product (630–1520 units of single-donor FFP of the same ABO blood group), manufactured at Octapharma AB (Stockholm, Sweden).	Filter and lyophilized in glass bottles. Reconstituted within 10–15 min with water for injection provided in a flexible bag.	Stored at room temperature for up to 2 years ABO-universal	Approved for prehospital transfusion regulatory assessment for marketing authorization in selected countries of the European Union.	Heger and Gruber 2022 [92]; Octapharma 2023 [96]
Pooled spray-dried plasma (Resusix) by Entegrion	Plasmapheresis solvent/detergent pathogen-reduced, pooled, tested for HIV, HAV, HBV, HCV, and B19 Parvovirus pooled (1000–1500 units).	A spray-drying technique where a stream of plasma is exposed to high-temperature nitrogen gas (55 °C) for 15 milliseconds. Reconstituted with citrate phosphate buffer before use.	ABO-universal; similar to that obtained by freeze-drying and within EU and FDA limits.	Phase I clinical trial completed in 2016.	Pusateri et al., 2019 [90]; Pusateri et al., 2016 [45]; Entegrion 2017 [97]
Single-donor spray-dried plasma (Frontline on-demand plasma (ODP) by Velico Medical	Single-donor plasma pretreated with glycine–hydrochloric acid and stored at 1 to 6 °C.	A prototype spray continuous-flow spray-drying in an open-system plastic disposable that was integrally attached to the finished product bag dryer by Velico Medical: one unit at a time at local blood centers. Reconstituted in 3.65 ± 0.44 min (n = 60) in water. Reconstituted in water (190 mL) and 1.5% glycine within 5 min.	Comparable global coagulation function PT, PTT and TEG, greater than 80% levels of functional coagulation factors and related proteins and chemistry analytes except for Factor XIII (74%) and 60% vWF/RiCoF. When reconstituted in water 25% recovery rates for FV and FVIII compared to FFP control were 58% for FVIII, 72% for protein S, and 75% for V; ABO type specific.	Approved for phase I clinical trial.	Pusateri et al., 2022 [22]; Liu et al., 2019 [98]; Booth et al., 2012 [61]; Velico Medical [99]

ACD-A, anticoagulant citrate dextrose solution A; CPD, citrate phosphate dextrose solution; EMS, Emergency Medical Service; FFP, fresh frozen plasma; HIV, human immunodeficiency virus; HLA, human leukocyte antigen; RT, room temperature; S/D, solvent/detergent; vWF/RiCoF, von Willebrand factor/ristocetin cofactor.

Some of these new- or next-generation dried plasma products have shown safety in early phase clinical trials. Octapharma’s FDP (OctaplasLG Lyo) from pooled S/D-treated plasma OctaplasLG has been approved for prehospital transfusion in Europe in February 2023 [96]. Teleflex’s FDP from single-donor, non-pathogen-reduced plasma was evaluated in a dose escalation trial in which 24 male or female healthy volunteers donated either whole blood or apheresis plasma, which was processed into FFP or FDP, and reinfused into the same donor 3 to 6 weeks later [93]. There were no significant differences in coagulation factor responses or in the adverse events of the subjects undergoing the autologous transfusion of up to 810 mL of FDP compared with the same amount of FFP in a crossover design. No differences between the FDP and FFP groups were observed in selected inflammatory or thrombotic markers. Velico Medical spray-dried plasma (ODP) was granted FDA approval to proceed with a phase I (human) clinical study in 2023 [99].

FDP is normally administered via a peripheral intravenous line. Intraosseous administration of FDP was feasible, but requires a high skill level [105]. A case report showed successful intraosseous transfusion of FDP (LyoPlas) for prehospital resuscitation of a 13-year-old girl suffering from severe hemorrhagic shock as a result of gunshots and a grenade blast [106].

## 4. In Vitro Characterization of Dried Plasma

Multiple metrics that include global and specific functional hemostatic measures, endothelial functions, residual cell counts, microparticle phenotypes have been used to assess the quality of different dried plasma.

### 4.1. Hemostatic Properties before and after Drying Process

#### 4.1.1. FLyP

The hemostatic properties of FLyP were measured by various in vitro coagulation assays including PT; activated partial thromboplastin time (aPTT); fibrinogen concentration; activity of Factor (F) V, FVIII, FXI, and FXIII; protein C; free protein S activity; antithrombin; and antiplasmin using a coagulation analyzer, Thrombelastograph (TEG) 5000. These properties were compared to the initial plasma (FFP) before lyophilization and treatment with amotosalen and ultraviolet light for pathogen reduction [69]. The PT ratio and aPTT were significantly increased by 8% and 11%, respectively, after plasma processing; however, there was no significant modification in TEG parameters (reaction time R, α angle, and maximum amplitude MA) between FFP and FLyP. FV and FVIII showed decreases in their activities by 25% and 20%, respectively, but remained within normal range (0.7–1.2 and 0.5–1.5 U/mL), while the activities of other factors (fibrinogen, FXI, FXIII, antiplasmin, antithrombin, protein C, and protein S) were preserved.

After lyophilization, the levels of two out of the nine hemostatic proteins (fibrinogen, FV, FVIII, FXI, antiplasmin, antithrombin, protein C, and protein S) in FLyP decreased significantly (FV: −25%, FVIII: −20%), but were within physiological concentration range and overall hemostatic efficacy was not affected [48]. Thrombin generation assays demonstrated preserved thrombin potential and the absence of coagulation factor activation during the lyophilization processing. Collectively, these TEG studies demonstrate that the in vitro hemostatic properties of FLyP do not differ from FFP. In addition, hemodilution studies using WB diluted with 30% Lactated Ringer’s solution and either 30% FFP or FLyP demonstrated clotting capacity similar to undiluted blood, suggesting that sufficient coagulation factors are still present in plasma to induce clot formation, even in a hemodiluted state.

#### 4.1.2. LyoPlas

Both the coagulation and anticoagulant factors (fibrinogen, FV, FVIII, FXI, von Willebrand factor/ristocetin cofactor (vWF/RiCoF) activity, protein S, antithrombin, plasminogen, and antiplasmin) of LyoPlas were determined by commercially available tests and adverse transfusion events were compared to the starting FFP [49]. Specifically, the coagulation factors’ levels in LyoPlas were within normal ranges, with decreases in some activities compared to its initial plasma. Only FVIII and vWF/RiCoF were reduced by more than 20% after lyophilization. FV, FXI, fibrinogen, antithrombin, and plasminogen were reduced by 5.1–11.1% after lyophilization. Protein S was not affected. vWF multimer patterns were investigated by gel electrophoresis and densitometry. Samples from two LyoPlas units did not show any alteration of vWF multimers compared to the corresponding plasma samples drawn before lyophilization. Investigation of a disintegrin and metalloproteinase with a thrombospondin type 1 motif, member 13 (ADAMTS13) that regulates vWF multimer length, did not show a relevant difference in activity before and after lyophilization among the six LyoPlas units tested. Hence, ADAMTS13/vWF analyses demonstrate normal primary hemostasis function after lyophilization.

The hemostatic capacity of WB reconstituted from LyoPlas was compared to that of WB reconstituted from FFP by mixing with aliquots of red blood cells and platelet concentrate at ratios of 1:1:2, 1:1:1, and 2:1:1 [107]. To achieve three different concentrations, LyoPlas was reconstituted in 50 mL (LP50), 100 mL (LP100), and 200 mL (LP200) of water. Hematocrit, platelet counts, and fibrinogen levels of the reconstituted WB at the three ratios were similar between the FFP and LP200 units, but were lower compared with the corresponding ratios in the LP100 and LP50 units. The activity of procoagulant (FII, FV, FVII, FVIII, FIX, FX, FXI, FXII, and FXIII) and anticoagulant factors (antithrombin and protein C) increased linearly with the increasing plasmatic fraction, and at 1:1:2 ratio, the procoagulant and anticoagulant activities were significantly higher in the LP50 units compared with the FFP and LP200 units. Thrombin generation was similar throughout the four plasma groups at any of the ratios.

An early version of LyoPlas was produced from pooled and S/D-treated plasma. After the addition of glycine (36.6 mmol/L), pH adjustment, and sterile filtration, aliquots of 200 mL of pooled plasma from approximately 1000 donors were transferred into sterilized glass bottles and spun while frozen below −30 °C. Lyophilization was performed in a freeze dryer controlling pressure and shelf temperature to obtain lyophilizates with less than 1% water. Proteomic characterization of the resultant FDP by differential in-gel electrophoresis (2D-DIGE) showed that lyophilization did not change any of 600 plasma protein spots (protein alterations in intensity, size, shape or location on 2D-DIGE), while pathogen inactivation by S/D treatment caused significant changes in 38 protein spots, including antitrypsin, antichymotrypsin, and antiplasmin [55]. The Pearson correlation coefficient of the averaged values of the normalized fluorescence intensities of the individual protein spots before and after lyophilization was 0.983.

#### 4.1.3. Bioplasma FDP

Limited information about the freeze-drying effect on hemostatic properties is available for Bioplasma FDP. One in vitro study showed that transfusion of Bioplasma FDP could mitigate the dilution effect of a hemoglobin-based oxygen carrier (HBOC-201) on coagulation as measured by TEG, PT, aPTT, and fibrinogen level in a model of resuscitation for hemorrhage and hemodilution [108].

#### 4.1.4. Teleflex FDP

The hemostatic properties of new-generation FDP products have been studied in comparison with their initial plasma before freeze-drying. Teleflex FDP was manufactured from a single-donor FFP unit derived from apheresis plasma or WB collection using a Magnum shelf freeze dryer (Millrock Technology, Kingston, NY, USA) [93]. Coagulation and chemistry parameters on 155 pairs of FFP and their derivative FDP units were compared. FDP had a slight prolongation of mean clotting times (PT, INR, aPTT, and thrombin time) and minimal or modest reductions in the mean content of fibrinogen, FII, FV, FVII, FVIII, FIX, FX, FXI, FXII, protein C, protein S, plasmin inhibitor, plasminogen, antithrombin, vWF activity, and vWF antigen, compared with the FFP control, with the largest decreases being seen in FXII activity, by 15.73%, and activated FVII (FVIIa), by 15.12% [93]. All the parameters were within approximately ±15% agreement (mean percent change) of the respective starting materials and within the clinical reference ranges, except for PT and INR which were slightly above the upper limits, while FII, FX, and antiplasmin showed activities slightly below the lower limits of the clinical reference ranges. However, all of the coagulation proteins were well within the 20% margin, defined for bioequivalence by the US FDA [109]. The in vitro characterization data of FDP displayed sufficient preservation of plasma parameters, and therefore supported advancing to first-in-man clinical safety testing [93].

#### 4.1.5. HemCon FDP

HemCon FDP produced from single-donor FFP showed retention of coagulation functions and factor activities as measured by 3% increase in INR, 13% increase in aPTT, 3% increase in FV, 14% decrease in FVIIa, 2% decrease in FVIII, no change in antithrombin, 12% increase in protein S, and 28% increase in prothrombin fragment 1 + 2 (F1+2) [100]. The retention for FVIIa, protein S, and F1+2 appeared to have higher variation, which was contributed by the variability of the analytical method. The test results for all clinical batches were within the acceptable reference range. The pH and osmolality were increased and decreased by 1%, respectively. Furthermore, single-donor lyophilized male-type O+ plasma with factor levels within the normal range was produced and reconstituted in buffer provided by HemCon (Portland, OR, USA). Reconstituted HemCon FDP was compared to corresponding single-donor FFP using measures of endothelial cell (EC) function in vitro cell culture and assays. The in vitro studies included the following: permeability and transendothelial monolayer resistance, adherens junction preservation, and leukocyte-EC adhesion [110]. The in vivo studies included measures of hemorrhagic shock-induced pulmonary vascular inflammation and edema in a mouse model [110]. The FDP demonstrated similar effects to FFP across all measures of EC function, reduced pulmonary injury, inflammation, and vascular leak, as well as potent protective effects on the vascular endothelium in vitro and on lung function in vivo following hemorrhagic shock. In addition, compared to the prelyophilization values, the FDP produced by HemCon from pooled porcine plasma by centrifugation of WB and freeze-drying also retained 84% FVIII activity, 100% FIX, and 93% antithrombin activity [64]. INR was prolonged by 9% and aPTT was prolonged by 13%. The FDP was reconstituted to its original volume with sterile water containing ascorbic acid for pH adjustment, for transfusion in a swine model of complex multiple traumas including extremity fracture, hemorrhage, severe liver injury, acidosis, and hypothermia. Swine resuscitated with FDP had equivalent or higher mean arterial pressures, and similar coagulation profiles, plasma lactate levels, and post-injury blood loss compared with those treated with FFP. Swine treated with FDP-RBC had significantly less blood loss and lower interleukin 6 levels than those treated with 1:1 FFP-RBC. The decreased blood loss suggests an interaction between FDP and red blood cells that is not reflected in routine coagulation measures and is worthy of further study. The blunted interleukin 6 response could be due to ascorbic acid in the reconstitution fluid that has been shown to decrease inflammatory mediators [111].

#### 4.1.6. Terumo BCT FDP

Terumo BCT is developing a system with which blood suppliers can produce FDP (referred to here as TFDP) from plasma frozen within 24 h of phlebotomy (PF24) or FFP plasma units from up to 10 donors [91]. Unlike FLyP and LyoPlas stored in glass bottles, TFDP is stored in a durable, light-weight plastic package suitable for field use. The pooling of FFP normalized factor variability in TFDP, and did not negatively impact product quality, with specific coagulation factor activity levels and protein concentrations (FV and FVIII, fibrinogen, and proteins C and S) staying within the clinical ranges before and after lyophilization. A significant decrease in FVIII activity between pre- and post-lyophilization was reported, although the overall percentage difference (−12%) was well under 20%. FV, protein C and S activities, and fibrinogen concentration were not altered by the lyophilization process. The PT and aPTT values showed a significant rise post-lyophilization, with only 4.1% and 4.6% increase, respectively, well within the acceptable range.

Using the Terumo BCT freeze-drying system, Canadian FDP (CFDP) was produced from Canadian-sourced plasma collected and pooled from 10 donors by Canadian Blood Services. We conducted in vitro studies of CFDP in comparison with its source plasma [58,59,94]. There were no significant differences in ROTEM variables (coagulation time (CT), clot formation time, α-angle, maximum clot firmness (MCF), and lysis index 30), coagulation and fibrinolysis profiles (PT, aPTT, fibrinogen, FV, FVIII, antithrombin, D-dimer, protein S), or levels of immuno-inflammatory mediators (cytokines, chemokines, and vascular molecules) between the source plasma and the reconstituted CFDP samples [58,59]. Furthermore, no significant differences were observed in FII, FXI, ADAMTS13, protein C activities or thrombin–antithrombin (TAT) complexes, activated complement factor 5 levels, or in osmolality, between Canadian FFP pools and derived CFDP, before and after freeze-drying [94]. Statistically significant differences in all other factors attributable to freeze-drying were limited to <15% in all cases, except for activated complement factor 3 (64%), with only FVIII, protein S, and antiplasmin exhibiting a >10% decline. PT and aPTT values were modestly increased after freeze-drying, but within an acceptable range. There was no significant difference in TAT or D-dimer levels before and after freeze-drying, suggesting no activation of coagulation or fibrinolytic pathways. A lack of coagulation-related activation was also suggested by the fact that the activity of coagulation factors, including FXI, did not increase, before and after freeze-drying.

#### 4.1.7. OctaplasLG Lyo

OctaplasLG Lyo is another new FDP product manufactured by Octapharma AB (Stockholm, Sweden) from pooled (630–1520 units) and S/D-treated FFP of the same ABO blood group (OctaplasLG) [92]. Biochemical testing confirmed that OctaplasLG and OctaplasLG Lyo showed comparable values for global coagulation parameters, coagulation factors and protease inhibitors, activation markers of coagulation and fibrinolysis, and important plasma proteins. Specifically, PT and aPTT were comparable between OctaplasLG, OctaplasLG Lyo, and FFP. Reptilase time and thrombin time for both OctaplasLG products and single-donor FFP units were within the reference range for plasma, with the exception of the thrombin time for OctaplasLG Lyo which was slightly below the reference range of 14–20 s. Coagulation factors’ (fibrinogen, FII, FV, FVII, FVIII, FIX, FX, FXI, FXII, and FXIII) activities and protease inhibitors’ (antithrombin, antitrypsin, C1-inhibitor, heparin cofactor II, plasminogen, and protein C) levels for OctaplasLG and OctaplasLG Lyo were comparable and within the reference range for plasma, except for protein S and plasmin inhibitor levels below the lower limit of the reference range for plasma. Levels of vWF/RiCoF and ADAMTS13 were similar in both OctaplasLG products. Moreover, FVIIa, TAT complex, F1+2, and D-dimer were tested as indicators of coagulation and fibrinolysis activation and were comparable and within the reference range for plasma as well. Thrombin parameters (peak thrombin and endogenous thrombin potential) and ROTEM parameters (EXTEM CT and MCF) were comparable between OctaplasLG Lyo and OctaplasLG. Mean concentrations of the major plasma proteins (including total protein, albumin, immunoglobulins (Igs), and lipoprotein[a]) were within the reference range for plasma and comparable between OctaplasLG and OctaplasLG Lyo. Finally, the complement proteins 1q, 3, and 4 were within the reference range for plasma for both OctaplasLG products. Factor H and I levels were comparable for both the frozen and freeze-dried forms, with significantly higher levels seen in FFP compared to OctaplasLG (*p* < 0.05). Overall, the comprehensive biochemical investigation study confirmed that the quality and biological function of OctaplasLG Lyo is not impaired by the freeze-drying step.

#### 4.1.8. Lab-Made FDP

Huebner et al. prepared FDP from 200 mL of pooled liquid plasma (pLP) that was frozen and lyophilized using a Labconco FreeZone freeze dry system (Labconco Corporation, Kansas City, MO, USA). In vitro TEG studies showed the comparable enhancement of coagulation and attenuation of tissue plasminogen activator-induced fibrinolysis between FDP and pLP, suggesting that FDP is a potential alternative to plasma resuscitation in the treatment of hemorrhagic shock [112]. Mass spectroscopy showed that most of the proteins had similar levels in pLP and FDP, with 19 out of 125 proteins (15%) showing changes greater than 1.5-fold between the two groups, while 15 out of 19 proteins had higher levels in FDP than in pLP. Of these 15 proteins, 4 have a known role in coagulation: thrombin-activatable fibrinolysis inhibitor, FXIII B chain, FXI, and protein C. Among the remaining proteins that had changes greater than or equal to 1.5-fold from pLP to FDP, only antithrombin is known to have a role in coagulation. Although there were significant differences in several of the coagulation proteins, the overwhelming majority were conserved during the freeze-drying process, as shown in the near-identical TEG results.

### 4.2. Hemostatic Stability of FDP during Storage

The hemostatic stability of a number of FDP products was investigated after storage under different conditions over time, from hours to years. These conditions varied in storage temperature from −25 °C to 60 °C or were uncontrolled in a field environment.

#### 4.2.1. LyoPlas

After storage at 2 °C to 8 °C for 24 months, LyoPlas showed ≥ 10% reduction from 98.9% to 88.4% in activity for FV, from 0.79 IU/mL to 0.8 IU/mL for FVIII, and from 60.7% to 57.6% for vWF/RiCoF [49]. All other factors remained relatively stable, showing decreases from 2.6 g/L to 2.4 g/L for fibrinogen, from 78.3% to 72.1% for protein S, from 0.91 IU/mL to 0.84 IU/mL for antithrombin, and increases from 81.7% to 82.2% for FXI and from 91.1% to 100.7% for plasminogen. Storage at room temperature (23 °C to 27 °C) for 24 months mainly affected fibrinogen, which decreased to 58% of its original concentration, from 2.6 g/L to 1.5 g/L. FV activity decreased by 25.8%, from 98.9% to 73.4%; FVIII by 15%, from 0.8 to 0.68 IU/mL; FXI from 81.7% to 77.7%; protein S from 78.3% to 63.2%; antithrombin from 0.91% to 0.78%; plasminogen from 91.1% to 93.6%; and vWF/RiCoF from 60.7% to 45.1%. As a consequence, aPTT was also prolonged, from 37.9 s to 39.3 s and 47.7 s after storage at 2 °C to 8 °C and 23 °C to 27 °C for 24 months, respectively. The activity of antiplasmin was only reduced from 82.4% to 81.6% after 15 months of storage at 2 °C to 8 °C. The activities of fibrinogen, FVIII and aPTT were further determined after storing LyoPlas at different temperatures over a period of 39 months. While being stable between 2 °C and 8 °C, the fibrinogen concentration decreased from 2.6 g/L to about 1.5 g/L after 39-month storage at 23 °C to 27 °C, and was reduced to 64% of its original activity after storage at 38 °C to 42 °C for 34 days. Storage at 56 °C for 12 days led to 100% loss of fibrinogen activity [49].

The hemostatic profiles of coagulofibrinolytic mediators (fibrinogen, FV, FVIII, FXI; proteins S and C; aPTT; antithrombin; vWF; and INR) of LyoPlas were evaluated under various controlled storage conditions (stored at 4 °C, 25 °C, and 40 °C for 6 and 12 months) and one uncontrolled field condition, after uncontrolled storage for 15 months [113]. Except for fibrinogen and FV, which showed decreases of 7.9% and 19%, INR, aPTT, factor levels (FVIII, FXI, protein C, protein S, antithrombin and vWF) were not significantly different between freshly supplied LyoPlas and those stored at 4 °C for 12 months. When stored at 25 °C, fibrinogen and FV decreased at 6 and 12 months by approximately 29% and 27%, respectively, and INR increased at 12 months by 15%; other factors remained the same. These data, along with the measured clinically insignificant INR values (1.05 and 1.21 measured after 12 months at 4 °C and 25 °C, respectively) as an indicator for clot formation, suggest a negligible effect of these storage conditions. However, at 40 °C, all the measurements showed changes at 6 and 12 months, except protein C which remained unchanged. Most coagulation factors and proteins (fibrinogen, FV, FVIII, FXI, vWF, antithrombin, protein S) were far below normal laboratory ranges, especially after 12 months of storage. Similarly, increases in INR and aPTT and decreases in the levels of all the factors except antithrombin, FVIII, protein C, and vWF were seen 15 months after storage under the field condition. However, the levels of most clotting factors (fibrinogen, FXI, FVIII, antithrombin, protein C, protein S, and vWF) were still within the normal laboratory range.

A further stability study under uncontrolled Israeli field conditions categorized as climate zone 2 (Mediterranean/Subtropical) suggested possibly extending the shelf-life of LyoPlas from 15 to 24 months [114]. Specifically, compared to freshly supplied LyoPlas from the manufacturer (tested within 3 months after production), a significant increase in INR, PT, and aPTT, and decreases in fibrinogen and FVIII, were found after storage in the field for 6, 12, 18, and 24 months post-expiry of LyoPlas. Significant decreases were also demonstrated in factor XI at 12, 18, and 24 months post-expiry, in protein C at 18 months post-expiry, and in FV at 24 months post-expiry. The TEG R value increased in the samples at 24 months post-expiry, and the MA value decreased in the 12 and 24 months post-expiry groups. No statistically significant changes were found in D-dimer, protein S, antithrombin, vWF, TEG K value, and Angle value, in the 6, 12, 18, and 24 months post-expiry groups, compared to those of freshly supplied LyoPlas.

The clotting factor activities and proteomic profile of FDP produced from pooled and S/D-treated plasma (the plasma preceding LyoPlas) was evaluated by 2D-DIGE and mass spectrometry, after 24 months of storage in a closed bottle under a moderate vacuum at 25 °C and 60% humidity [55]. PT was increased to 38.30 s, slightly above the normal range of 26–37 s. The clotting factor activities were determined using the Clauss method for fibrinogen; chromogenic assays for FVIII, FXIII, antiplasmin, antithrombin, and protein C; and clotting assays for FII, FV, FVII, FIX, FX, FXI, FXII, and protein S. At the end of this storage period, the activities of 10 of the 14 clotting factors and inhibitors (fibrinogen, FII, FV, FVII, FVIII, FIX, FX, FXI, FXII, FXIII, antiplasmin, antithrombin, protein C, and protein S) were within the normal range. FVIII and FXI activities were decreased to 65.50% and 69.30%, slightly below the normal range (70–120%). The activities of antiplasmin and protein S were significantly decreased to 51.20% and 25.00%, respectively, displaying typical effects of the S/D pathogen inactivation procedure. In comparison to FDP without S/D treatment, antiplasmin and protein S activities were not altered. The proteomic analysis indicated a reduction in 38 of the 600 plasma proteins due to the S/D treatment and no changes to the plasma proteome resulting from the lyophilization.

#### 4.2.2. Terumo BCT FDP

When stored at 4 °C for 24 months, the PT and aPTT values of TFDP increased by 17.6% from 14.2 s to 16.7 s, and by 16.2% from 30.8 s to 35.8 s, respectively [91]. When stored at 25 °C for 24 months, these values increased by 27.5% to 18.1 s, and by 35.1% to 41.6 s, respectively [91]. The corresponding FFP showed 23% and 15% increases in PT and aPTT. The FV activity decreased to 44% in TFDP stored at 25 °C and 49% in those stored refrigerated, which were both outside of the clinical range (70–120%), as FV levels were already outside of the clinical range in the initial FFP (64%). When stored for 24 months at 25 °C, FVIII activity in TFDP significantly decreased compared to the FFP controls stored at −20 °C for the same time period (−28%). The FVIII activity was less pronounced in TFDP refrigerated for 24 months (−15% from FFP control). The fibrinogen concentrations in TFDP remained within 20% of the concentration in FFP throughout 24 months of storage, showing 2.62 g/L (−8% from FFP control) when refrigerated and 2.31 g/L when stored at 25 °C (−19% from FFP control). The protein C activity in TFDP stored for 24 months at 25 °C was 75% (+27% from control), and 79% (+34% from control) in TFDP refrigerated for 24 months, remaining within the clinical range (70–130%). The protein S activity in TFDP stored at 25 °C for 24 months decreased from 92% to 88%, and decreased from 92% to 84% when it was refrigerated for 24 months.

#### 4.2.3. Lab-Grade FDP

Dufresne et al. produced FDP from plasma samples centrifuged from EDTA WB donated by 30 male and female subjects. Plasma was aliquoted in 225 μL volumes and frozen to −80 °C and then rapidly placed in a Labconco freeze dryer (Labconco Corporation, Kansas City, MO, USA) with the condenser maintained at −90 °C with a 30 L per minute pump for 24 h before rapidly resealing the vials [115]. The lab-made FDP was stable at room temperature in a desiccator for at least 1 year as determined by liquid chromatography and tandem mass spectrometry. Protein analysis using sodium dodecyl-sulfate polyacrylamide gel electrophoresis with Western blot showed no release of tryptic peptides from complement component 4B, suggesting no plasma degradation. Although the FDP was not produced for transfusion, the study provided a sensitive method that indicated how the highly soluble proteins in human plasma may be preserved by freeze-drying with long stability and entirely reconstituted upon the addition of water.

#### 4.2.4. Other FDP Storage Stability Studies

The stability of coagulation factors in lyophilized plasma was also assessed under extreme temperatures at −25 °C [116] and 60 °C [117], respectively. The former demonstrated that there was no statistically significant change in functional activity for fibrinogen, FII, FV, and factors VII through XII in the lyophilized plasma when stored unreconstituted at −25 °C for one year, suggesting its use as a stable standard reference for the assay of coagulation factors. The latter study showed no change in PT after as long as 24 h of heating, with only a mild increase after 48 h and 72 h. FV activity decreased most significantly after heating (60% after 8 h and 48% after 24 h), but the activities of FVII, FVIII, and FIX exhibited minimum deviations from their unheated levels.

Interestingly, biochemical analyses of 30-year-old FDP demonstrated that the process of lyophilization and storage does not seem to cause any great changes in plasma components, including hormones, enzymes, and proteins [118]. In addition to this, our analyses of 80-year-old Canadian freeze-dried serum showed intact albumin, antithrombin, plasminogen, protein C, and protein S activity, as well as 71 proteins, the most prominent being albumin. The serum was also shown to be positive for hepatitis B by serological testing [57].

### 4.3. Hemostatic Stability of FDP after Reconstitution

Compared to the effects of freeze-drying and storage of FDP, the hemostatic stability of reconstituted FDP has been less studied.

#### 4.3.1. FLyP

After storage at 4 °C for 24 h, reconstituted FLyP showed decreases in FV of 38% from 65.2% to 40.2%, FVIII activity increased by 17% from 0.6 to 0.5 U/mL, fibrinogen by 10% from 2.7 to 3.0 g/L, increases in PT of 5.6% from 71.2 to 67.2%, and aPTT of 8.9% from 41.5 to 45.2 s [48]. A minimal reduction in PT (64.8% at 6 h vs. 67.2% at 24 h after reconstitution) was observed, which was explained by a partial activation of factor VII. In comparison, the corresponding changes were decreases of 37% for FV, 33% for FVIII, 10% for fibrinogen, and increases in PT of 14% and by 15% in aPTT, after storage for 24 h at room temperature. Storage at 4 °C after reconstitution ensures better preservation of clotting properties than storage at room temperature.

#### 4.3.2. LyoPlas

To determine its stability after reconstitution with sterile water following manufacturer’s instruction, four units of LyoPlas (one of each ABO blood group) were tested after 0, 3, 6, 9, 24 and 48 h at 21 °C to 22 °C for aPTT, fibrinogen, FV, FVIII, FXI, vWF/RiCoF, antithrombin, and protein S [49]. FVIII and protein S decreased within 48 h to 50% and 66% of their starting activity, while in the first 6 h there was only a loss in the 10% range, suggesting that LyoPlas is best used within 6 h after reconstitution when stored at room temperature.

The activities of coagulation factors (fibrinogen, FII, FV, FVII, FVIII, FIX, FX, FXI, FXII, and FXIII) and inhibitors (antithrombin, plasmin inhibitor, vWF antigen, protein C, and protein S) of pathogen-inactivated pooled FDP (preceding version LyoPlas) from different lots were evaluated over 6 days after reconstitution and storage at 4 °C [119]. The clotting activities of reconstituted LyoPlas were found to decrease by between −6.3% (FXI) and −14.9% (FVIII) after 6 h of storage at 4 °C and by between −6.3% (fibrinogen) and −24.3% (FVIII) after 6 days at 4 °C [119]. In comparison to baseline values, at 0 h, storage at 4 °C for 6 h led to a decrease in the activity of FVIII of 14.9%, FIX decreased by 6.9% and FXI by 6.3%, and an increase in the activity of plasmin inhibitor of 10.2%, while no change was observed in fibrinogen. The changes increased over the storage time. Storage for up to 6 days resulted in a further decrease in the activity of FV of 15.0%, FVIII decreased by 24.3%, FIX by 13.4%, FXI by 22.9%, fibrinogen by 6.9% and plasmin inhibitor by 17.5%. If we define a deviation of ≤5% from baseline as unchanged, the other factors and inhibitors (FII, FVII, FX, FXII, FXIII, vWF antigen, antithrombin, and protein S) remained almost unchanged over time, except for protein C which had a 8.2% increase in activity. The activity of all measured coagulation factors (fibrinogen, FII, FV, FVII, FVIII, FIX, FX, FXI, FXII, and FXIII) and inhibitors (antithrombin, plasmin inhibitor, vWF antigen, protein S, and protein C) in a time course of up to 6 days met the required quality standards set by the manufacturer for FDP, which consist of individual coagulation factor activity of ≥50%, antithrombin activity between 70 to 140%, and fibrinogen levels between 1.0 to 3.0 g/L.

Our in vitro study of the long-term (≥168 h) hemostatic stabilities of reconstituted LyoPlas showed decreased stability in both global and specific hemostatic profiles with increasing storage temperatures, particularly at 35 °C, where progressive changes in ROTEM CT and MCF, PT, aPTT, fibrinogen concentration, FV, antithrombin, protein C, and protein S activities were seen even after storage for 4 h [120].

#### 4.3.3. Canadian FDP

We also conducted an in vitro study of the short-term (≤24 h) hemostatic stabilities of CFDP after reconstitution and storage under different conditions [120]. When compared to the initial reconstituted CFDP, there were no changes in ROTEM measurements for INTEM MCF, EXTEM CT and MCF, or the Stago measurements for PT, aPTT, D-dimer concentration, plasminogen, and protein C activities after storage at 4 °C for 24 h and room temperature for 4 h. However, an increase in INTEM CT and decreases in fibrinogen concentration, FV and FVIII, and protein S activities were observed after storage at 4 °C for 24 h, while an increase in FV and decreases in antithrombin and protein S activities were seen after storage at room temperature for 4 h. The short-term stability of CFDP in global hemostatic properties after reconstitution and storage at room temperature is consistent with the shelf life of reconstituted LyoPlas [49].

In summary, the post-reconstitution hemostatic stability of FDP products would decrease over time with increasing storage temperature, with a significant loss of hemostatic functions at 35 °C compared to 22 °C or below. Therefore, the shelf life of reconstituted FDP should be recommended according to the storage temperature.

### 4.4. Spray-Dried Plasma (SDP)

In addition to lyophilization, dried plasma has been prepared using the spray-drying method and characterized for proteins and coagulation activities [61,74]. The spray-drying method rapidly removes water using a combination of heated, clean dry air, variable plasma flow rates, pressure, and specialized spray nozzles to atomize plasma and rapidly remove the water fraction, down to about 1%. Compared to freeze-drying, spray-drying enables rapid formation of very small particles and faster production, using desk-top equipment that is suitable for many facilities [61]. In addition, spray-drying could result in a five-fold improvement in protein viability compared with freeze-drying [121]. However, spray-drying simultaneously exposes the proteins to high pH and heat, which are recognized influential factors of protein instability [98]. Moreover, the spray-drying method is less commonly used for the production of dried plasma for transfusion.

Currently, two SDP products are under development: a single-donor SDP (ODP by Velico Medical [98] and a pooled (1000–1500 units) S/D pathogen-reduced SDP (Resusix by Entegrion) [97]. Only the effects of the drying process on hemostatic properties have been assessed in vitro and animal models. No clinical trials for safety and efficacy of SPD have been reported.

#### 4.4.1. Velico Medical ODP

Specifically, even with significant losses in the activities of key coagulation factors (18% for fibrinogen, 17% for FVIII, and 26% for FXIII), ODP is comparable to its paired FFP in global coagulation function, as assessed by aPTT; in clot rate, as measured by angle (α); and in clot strength, as measured by TEG MA [98]. Compared to FFP, ODP had a 10% increase in PT, a 49% increase in thrombin time, and a 32% increase in TEG clot time, which may be attributed to the reduced pH in ODP. In addition, ODP had greater than 80% levels of functional coagulation factors (fibrinogen, prothrombin, V, VII, VIII, IX, X, XI, and XII) except for FXIII which showed 74% activity, and vWF/RiCoF with 60% activity relative to FFP, and inhibitors (antithrombin, protein C, and protein S, plasminogen, plasmin inhibitor, C1 esterase inhibitor, and alpha 1-proteinase inhibitor). ODP and FFP were similar in ADAMTS13 activities, coagulation activation as measured by F1 + 2, TAT complex, and D-dimer. In complement activation, ODP was similar to FFP in the C3a level, but slightly elevated in C5a (9.6 vs. 7.3 ng/mL). ODP was similar to FFP in total protein and comparable to FFP in the levels of citrate, albumin, calcium, Igs, lipoproteins, and lipids (94–105%). ODP was significantly different from FFP in pH (7.03 vs. 7.62) and osmolality (394 vs. 309 mOsm/kg) as a result of the pretreatment with glycine–hydrochloric acid (GlyHCl) solution.

Further study showed that pre- and post-treatment with 20 mM GlyHCl and 50 mM glycine in the spray-drying process could mitigate the cleavage of high-molecular-weight vWF multimers and result in more than 69% vWF/RiCoF activity in ODP, compared to only 36% activity in untreated ODP [122]. The treatment with 20 mM GlyHCl and 50 mM glycine led to a significant increase in platelet adhesion to collagen under normal arterial shear conditions compared to FFP, perhaps due to the increased number of smaller vWF multimers in ODP despite a diminution in high-molecular-weight multimers of vWF, which is a known effect of the spray-drying process [122]. When combined with RBC and platelets to reassemble WB, the treated ODP showed a small but significantly lower MA and a higher LY30 than FFP in TEG results, although both were within normal ranges. There were significant differences in thrombin generation between the treated ODP and FFP. The total number of particulates was much greater in FFP than in the SDP, with the majority of these being of platelet origin. In vitro testing of platelet adhesion and thrombus formation using a collagen-coated microfluidic channel to simulate bleeding in vivo showed that WB samples, reconstituted with either ODP or frozen plasma, had similar platelet adhesion and overall clot characteristics. These data support the development of clinical studies to evaluate the efficacy and safety of ODP in trauma patients [123].

Previously, SDP was also produced from plasma separated by centrifugation and a manual plasma extractor, as per standard FFP procedure, from 500 mL of WB collected by standard venipuncture techniques from single randomly selected research donors of blood group O rhesus factor D positive [61]. Leukofiltration was not performed. The plasma was spray-dried into small microparticles using a spray dryer composed of an atomizer, gas disperser, drying chamber, and systems for gas and moisture exhaust and powder recovery. Reconstitution of 3 g SDP was achieved with 30 mL of 1.5% glycine or deionized water within 5 min. Compared to the matched FFP, there was no significant intergroup difference in recovery for total protein, albumin, IgG, IgA, or IgM (96% or higher). Except for FVIII, which exhibited a recovery rate of 58% due to its heat lability, the recovery rates of clotting factors and inhibitors (fibrinogen, FV, FVII, FIX, vWF, ADAMTS-13, protein C, protein S) in the glycine reconstituted products ranged from 72% to 93% [61]. In comparison, reconstitution with deionized water yielded significantly lower recovery rates of 25% for FV and FVIII, and 52% to 92% for the remaining factors, compared to FFP controls [61].

#### 4.4.2. Entegrion Resusix

In addition to single-donor SDP, a pooled SDP product called Resusix was produced by Entegrion Inc. (Research Triangle Park, NC, USA) from large pools of plasma following the US FDA regulations, frozen within 4 h, and pathogen reduced by S/D treatment. The pooled and S/D-treated SDP showed greater thrombin generation; a smaller number of residual cells; a negligible number of microparticles, likely due to their removal by S/D treatment; and higher total thrombin formation compared with FFP and liquid plasma (LP) [62], thereby raising the potential for thrombotic issues. When SDP and its initial S/D-treated plasma were compared, lower thrombin peak values were observed, suggesting further study on the clinical implications of altered thrombin generation kinetics in the SDP. Furthermore, the Resusix SDP reconstituted in a pH-adjusted phosphate buffer showed the equivalence of FFP and S/D-treated FFP (the starting material for SDP) on the modulation of endothelial function and inflammation in vitro and in vascular endothelial cells [124,125]. In addition, SDP and FFP equally corrected base excess to normal levels; modulated pulmonary vascular permeability and lung inflammation, as indicated by reduced alveolar wall thickening; leukocyte infiltration; and breakdown of endothelial cell junctions in a mouse model of hemorrhagic shock [125]. McDaniel et al. compared FFP and SDP for their effects on blood pressure, cerebral blood flow, blood–brain barrier (BBB) integrity, and markers of endothelial cells and tight junction proteins, in a rat model of controlled cortical impact brain injury and 25% blood loss [126]. SDP was reconstituted to one-third of its original volume of FFP. Resuscitation was carried out with either an equal blood loss volume of FFP or an 80% blood loss volume of reconstituted SDP. The resuscitation with FFP and SDP had similar influences on cardiovascular physiology and cerebral perfusion, and SDP could be superior to FFP in reducing post-injury BBB compromise and protecting vascular endothelia [126]. These findings suggest that SDP may be a suitable alternative to FFP in traumatic brain injury patients who require plasma resuscitation [126].

#### 4.4.3. Assessing Drying Effects of FDP and SDP Using Porcine FFP

To assess the drying effects, FDP and SDP were also produced from porcine FFP in a lab setting using a Virtus Unitop 600 SL lyophilizer [127] and a Buchi Mini Spray Dryer B-290 (BUCHI Labortechnik AG, Postfach, Switzerland), respectively [128]. In vitro testing revealed that both drying processes could preserve PT/aPTT and clotting factors (fibrinogen, FII, FVII, FIX, protein C and protein S), but they resulted in an alkaline pH (>8.2) upon rehydration, and were titrated with 20 mmol/L glycine to obtain a final pH = 7.4 [127,128]. Infusions of the porcine FDP and FFP (instead of human FDP and FFP to avoid potential xenogeneic reactions) were equally effective in correcting the coagulopathy as indicated by PT/INR, TEG values, in the swine model of multiple injuries (femur fracture and grade V liver injury) and hemorrhagic shock (50% to 70% blood loss) [127]. In addition, the coagulation profiles of FFP and SDP rehydrated with the original volume of sterile water were similar, with SDP rehydrated with one-third of the original volume showing a prolonged PT/aPTT and increases in the activity levels of clotting factors (fibrinogen, FII, FVII, and FIX) and antithrombotic protein S, but a decrease in protein C activity. In a multiple injuries/hemorrhagic shock swine model, SDP was only rehydrated with one-third of the original volume of water for infusion and was as effective as FDP and FFP in maintaining survival and reversing trauma-associated coagulopathy, as indicated by the recovery of PT/INR and TEG MA to near-baseline levels [128]. Porcine SDP was also produced using the same spray-drying process as human SPD (Entegrion, Inc., Durham, USA) and rehydrated to its original volume with sterile water (yielding an isotonic preparation). Additionally, 20 mmol/L of glycine (pH 2.40) was used to adjust pH to 7.4. In a clinically relevant lethal swine model of rib fracture and grade VI liver injury, administration of the SDP significantly improved survival and prevented the development of coagulopathy without any long-term organ dysfunction or complications during the study period of 7 days [129].

## 5. Discussion

Early plasma transfusion within minutes of injury reduces mortality and is superior to crystalloids or colloids for the treatment of hemorrhagic shock in prehospital conditions [87]. Transfusion using blood products is an integral part of prehospital damage control resuscitation, and continued advancements are necessary to improve patient outcomes.

FFP and liquid plasma present several constraints, including the need for storage and transportation conditions, trained personnel, and additional thawing time before administration [33]. These limitations pose challenges for their use in remote locations requiring long transport times or austere environments requiring prolonged extrication [33]. The solution is to have a product that is readily available, easy to store and transport, and can be administered quickly and safely. Dried plasma provides all these advantages, with a shelf life of up to two years at room temperature, and reconstitution with water for transfusion within minutes. FDP has been shown to be an appropriate alternative to FFP, with all of the benefits of its source plasma [22,130], including the following:

- Physiological relevance, thereby preventing hemodilution and acidosis;
- Provision of coagulation factors and reduction in hyperfibrinolysis;
- Correction of the endotheliopathy of the trauma;
- Reduction in intestinal permeability;
- Reduction in metabolic derangements;
- Provision of logistical advantages in scenarios when storing and thawing frozen plasma is not feasible and rapid plasma transfusion is required, such as prehospital and battlefield conditions.

FDP is recommended to replace in-theater liquid and frozen plasma when there are significant logistical issues, such as in austere combat environments, remote rural settings requiring long prehospital transport times, or when speed of use/availability is an important factor [30]. However, the beneficial effect of existing FDP products on clinical outcomes may not be as high as initially anticipated when tested in randomized, multicenter clinical trials [80,81]. On the other hand, the logistical advantages and clinical benefits of FDP may be insignificant in urban regions, with short transport time.

In the US, FDP is not currently approved by the FDA, except for limited emergency authorization use of French FDP (FLyP) by the US military when FFP is not available or practical [53,54]. Although it is approved in European countries, no recommendation in favor or against the prehospital use of FDP can be provided in European guidelines on the management of major bleeding and coagulopathy following trauma [131]. The French doctrine for tactical combat casualty care recommends the use of FLyP for bleeding combat casualties as early as possible when cold-stored low-titer group O WB is not available [132]. According to the South African society of anesthesiologists perioperative patient blood management guidelines [73], Bioplasma FDP has the same indication as FFP and can be used as a substitute for FFP. At sites with no whole blood, FDP is commonly used for traumatic hemorrhage resuscitation on its own, independent of other blood products, and as the plasma component of 1:1:1 balanced resuscitation.

There are only a few manufacturers of FDP in the world. When coupled with the need for plasma donations as the substrate for production, this further limits the availability of FDP [45]. FDP from type AB plasma is considered a universal donor as the plasma from AB donors does not contain antibodies; therefore, it is best suited for use at the point of injury. However, the low prevalence of AB blood donors further limits the product’s availability [104]. Broader availability of dried plasma appears to be also limited by product-specific, business-related, and regulatory issues [90], and is dependent on centralized manufacturing for currently licensed FDP products [74]. Both Terumo BCT FDP and Velico Medical SDP are manufactured using established regional blood centers. The advantages and disadvantages of centralized and decentralized manufacturing models have been discussed by Popovsky and While [74].

FLyP and LyoPlas are the most extensively studied dried plasma in both in vitro and in vivo, and in clinical trials. The new- or next-generation of Teleflex FDP has shown to be safe in an early phase clinical trial [93] and Velico Medical SDP, which has promising preclinical profiles, is embarking on its first-in-human trials [99]. Compared to freeze-drying, spray-drying has not been used much to produce dried plasma for eventual reconstitution for transfusion, although both have demonstrated feasibility and logistical advantages over FFP and liquid plasma.

All known drying technologies (freeze- and spray-drying) have some impact on procoagulant or anticoagulant protein activities, particularly those known to be labile. [45]. Commonly recognized deleterious factors inherent in the drying process of protein molecules are adsorption, shear, and thermal and dehydration stresses, which can result in protein unfolding, aggregation, and denaturation [133]. In addition, the drying process may cause an increase in plasma pH due to the evaporation of carbon dioxide [69,134] and acids (ascorbic acid, citric acid, glycine–hydrochloric acid) may be added to dried plasma or reconstitution solution to adjust the pH for transfusion in animals [64,98,135]. The optimal solution for buffering lyophilized plasma in humans is unknown and will require further investigation. For now, sterile water is used to reconstitute dried plasma for clinical transfusion.

The prerequisite for the use of dried plasma for transfusion in bleeding patients is that hemostatic functions are conserved. The FDA has informed future manufacturers of dried plasma that a minimum of 80% activity of defined coagulation proteins must be retained in the final product, considering the wide, natural variability of biologics [136]. Alternatively, FVIII is a sensitive factor, routinely used as the quality marker of plasma, as quality control demands that FVIII is above 70% of the value of the freshly collected plasma unit (Council of Europe, 2005) [137]. The German guidelines for manufacturing blood components require a plasma FVIII activity of at least 70% of baseline values when measuring single units [138].

Therefore, in vitro studies on dried plasma have been focused on the effect of the drying process on global coagulation and activities of key clotting factors and inhibitors. These include PT, PTT, and fibrinogen, which is often depleted in bleeding patients, as well as FVIII and vWF, which are classical indicators of plasma quality. FV and FXI activities were also measured because patients with deficiencies of these factors are treated with plasma. Antithrombin and antiplasmin were included as they represent important regulatory coagulation factors. Protein S is known to be affected by S/D treatment and decreases when plasma is stored at 4 °C and room temperature for days [49,55,139]. TAT complexes and D-dimer levels were measured for the activation of coagulation and fibrinolytic pathways [92,94,98] and FVIIa and F1+2 were also markers for coagulation activation [92,98]. In addition, complement proteins (1q, 3, 4), factors (C3a, C5a), and antibodies (Igs A, G, M) [92,94,98], as well as immuno-inflammatory mediators (cytokines, chemokines, and vascular molecules) were analyzed [58]. Collectively, testing global coagulation and activities of these coagulation proteins is important to ensure the functional efficacy of dried plasma products.

The impact of both freeze- and spray-drying processes on reducing coagulation factors’ activities has been well studied. Overall, the prevalent evidence suggests that both FDP and SDP preserve the majority of coagulation and fibrinolysis factors above 80% activity, with statistical decreases in FV, FVIII, and vWF activities of more than 20%, and slight increases in PT and PTT by less than 10%. As summarized in Table 2, the most commonly measured coagulation factors (fibrinogen, FV, FVIII, FXI, antiplasmin, antithrombin, protein C, and protein S) lost activities, to various extents, due to the drying process. Relative to the source plasma levels, activities were reduced, at most, by 30% for FV and 20% for FVIII in FLyP [48,69], 22% for FVIII in LyoPlas [49], 15% for FV in Teleflex FDP [93], 21% for FV [59] and 14% for FVIII in CFDP [94], 13% for FVIII in TFDP [91], 14% for FVIII and FXI in OctaplasLG Lyo [92], and 18% for fibrinogen in ODP [98]. vWF/RiCoF was reduced by −25% in LyoPlas [49] and −40% in ODP [98]. Other factors, such as antiplasmin, antithrombin, protein C, and protein S display less than 10% changes by the drying process. The decreases in certain clotting factor activities of reconstituted FDP are comparable to the expected loss from the thawing process for thawed plasma or S/D-treated plasma [64,140]. The losses in some factor activities by the S/D treatment were greater than those by drying [55]. On the other hand, immediately after the thawing of FFP, there could be significant decreases in fibrinogen, FV, antithrombin [141]. Importantly, both in vivo hemorrhage models and clinical experience have shown the safety and efficacy of dried plasma in comparison to its source plasma [32,64,93,110,128,129].

In contrast, spray-drying resulted in more activity reduction with losses of approximately 14% for fibrinogen, 21% for FV, 42% for FVIII, 25% for vWF, and 28% for protein S activity when reconstituted in 1.5% glycine. Even higher activity losses (e.g., approximately 75% for FV and VIII) were seen when the plasma was reconstituted in water [61]. The improved process could retain greater than 80% levels of functional coagulation factors and related proteins and chemistry analytes except for FXIII (74%) and vWF/RiCoF activity (60%) [98].

The variations in the hemostatic profiles of dried plasma products are likely related to their source plasma, which may have different qualities [62] and a high correlation in activities with the resultant FDP [142]. Never-frozen liquid plasma was found to have a better coagulation profile and factor activity when compared to FFP [143]. Compared to the source FFP, standard S/D treatment causes a decrease in vWF activity (24%), fibrinogen (16%), factor V (37%), factor VIII (22%), protein S (44%), and alpha-2 antiplasmin (79%) [144]. Similarly, amotosalen with UV light reduces fibrinogen (35%), factor V (23%) and factor VIII (33%), but has minimal effects on vWF, antiplasmin, and protein S [144]. A newer S/D treatment product, Octaplas LG (Octapharma, Lachen, Switzerland), received FDA clearance in 2013. It employs a prion reduction step and a modified S/D process that better preserves factor levels [145]. FDP produced from S/D-treated plasma (OctaplasLG Lyo) showed lower protein S and antiplasmin activities due to the S/D treatment [146], as well as lower FV, FX and vWF functions. FDP produced from pooled plasma units resulted in lower standard deviations in factor activity levels compared to that from an individual plasma unit [91].

The FDA does not require specific quality parameters or criteria for various plasma products (FFP, liquid plasma and S/D-treated plasma), but rather licenses the plasma processing method. Conversely, the European Union has established plasma criteria, including pH (6.5–7.6), osmolality (minimum of 240 mOsm/kg), total protein content (minimum of 45 g/L), absence of irregular RBC antibodies, hepatitis A virus antibody levels, and coagulation factor concentration ranges. The coagulation factor criteria include FVIII, FV, FXI, (>0.5 IU/mL), protein C (>0.7 IU/mL), and antiplasmin (>0.2 U/mL) [62].

More FDP products have been developed in comparison to SDP. The long-term stability of SPD has not been examined. In comparison (Table 2 and Table 3), freeze-drying and spray-drying may have different effects on clotting factors and inhibitors; e.g., spray-drying shows more reduction in fibrinogen concentration (−18%), FXIII (−26%) and vWF/RiCoF activity (−40%), similar reduction in FVIII, and a positive effect on FV activity (6%) [98].

On the other hand, spray-drying has already been used successfully in the processing of food and pharmaceuticals [147]. Spray-drying is a very fast and technically easy to scale up in a cost-effective fashion [148], and also results in a five-fold improvement in protein viability compared with freeze-drying [121]. Compared to freeze-drying, which yields powdered chunks, spray-drying yields a fine powder that is much easier to reconstitute and may inactivate infectious materials by controlled heat [129]. Freeze-drying is more suitable for thermo-sensitive proteins than spray-drying, which involves hot-air drying exertion [149].

Stability studies on FDP products (LyoPlas and TFDP) indicated increases in PT and PTT, and decreases in the levels of specific coagulofibrinolytic mediators (fibrinogen, FV, FVIII, FXI, vWF, antithrombin, protein C, and protein S) over time when stored in a dry state under different temperature-controlled and uncontrolled field conditions [49,91,113,114,150]. These changes increased with storage temperature, but most global and specific hemostatic functions were within clinical ranges when stored at 4 °C and room temperature for up to two years [49,91,113,114,150], suggesting that dried plasma is stable for more than one year at room temperature without affecting safety or efficacy. Plasma stability was defined as the period during which there was a change of less than 10% from the initial value [151].

The hemostatic stability order from the least to most for LyoPlas is as follows: FVIII, fibrinogen, FXI, protein C, FV, vWF, protein S, antithrombin, D-dimer when stored in uncontrolled field conditions [114]. However, in another study by the same group [113], FV and protein C showed the most and least changes when stored under different temperatures. This discrepancy could be due the difference in the initial FV level of freshly supplied FDP used in the two studies (72.66% [113] vs. 107.38% [114]) and different storage conditions. Different extents of changes were also observed between FDP products. LyoPlas showed smaller decreases in FV and FVIII activities (26% and 15%) [49] compared to TFDP (44% and 28%) [91] after storage for two years at room temperature. In contrast, a larger decrease in fibrinogen concentration was seen in LyoPlas than TFDP (46% vs. 20%).

In addition to the stability of FDP in a dry state, it is important to examine the stability of FDP after reconstitution for optimal use in prehospital settings, which can be influenced by field conditions in particular storage temperature and time. Our results confirmed that the hemostatic properties of reconstituted FDP were not altered at 4 °C or room temperature for a short duration (<24 h), but long-term stability over a range of storage conditions was reduced and is worth further investigation.

Global coagulation functions and key coagulation and fibrinolysis factors were affected by storage temperature differently, beginning to change or lose their activities at different storage times after reconstitution [48,49,119,120]. In addition, each clotting factor activity may be affected differently by the drying and storage. It appears that fibrinogen stability, relative to other factors, was more affected during storage than freeze-drying. Furthermore, the dried and reconstituted FDP showed different changes in hemostatic stabilities when stored under various conditions over time. Factor VIII was less stable than factor V in FDP, but showed the opposite after reconstitution [49,113]. Protein S was mainly affected by storage after reconstitution [120].

Overall, FVIII and vWF/RiCoF are the two least-stable factors, with significant losses in activities after the drying process [49,94], storage at room temperature for 24 months [49,91], and within 48 h after reconstitution [48,91]. This is consistent with the literature, which shows that FVIII is the worst-affected factor with typical losses of 30–40% after storage for 5 days following thawing of frozen plasma [152]. vWF is shown to be the most impaired factor, followed by FVIII in liquid plasma when stored at 1 to 6 °C, over 30 days [153]. FVIII interacts non-covalently with vWF in plasma, protecting FVIII from degradation and prolonging its half-life [154]. If vWF loses activity, more FVIII is free, and may also lose activity in a synergistic manner.

Despite a certain level of factor reduction (20–25%), both freeze- and spray-drying have not been shown to profoundly alter the in vitro hemostatic efficacy of plasma, as shown by the less than 10% of changes in PT, PTT, and viscoelastic hemostatic assays.

While it has been standard practice to measure specific factors or proteins between products as a reflection of efficacy, global and functional measures of hemostasis such as viscoelastic hemostatic assays have also been used with the assumption that these assays reflect in vivo function more accurately [58,69]. Other emerging technologies include proteomics based on mass spectrometry analysis for in-depth protein quantification [55,112].

The discrepancies in the effects on hemostatic properties could be due to differences in measurement protocols, including the instruments and reagents used for each test, storage temperature and time, and the type of FDP-producing plasma (single-donor or pooled or S/D-treated plasma). For example, the reduced stability of protein S in our study of reconstituted CFDP and LyoPlas could be due to the difference in the assays used to detect protein S (activity versus antigen methods) [120]. FV and FVIII showed greater stability, while protein S showed reduced stability in S/D-treated plasma [139,155] and FDP [55], respectively. FV and FVIII showed more reduced stability in FFP than in S/D-treated plasma, while protein S showed greater stability in FFP during 48 h after thawing, storage at both 4 °C and room temperature [139], and storage at 20 °C for 5 days [155]. After 24 months of storage at 25 °C, nearly all the clotting factors in S/D-treated FDP retained their activities except for plasmin inhibitors and protein S, while FDP without S/D treatment did not show these reduced plasmin inhibitors and protein S activities [55].

In addition to the hemostatic properties, another important measure of plasma efficacy and quality may be its ability to protect or restore endothelial function during traumatic hemorrhage [156]. Only a few studies showed equivalent modulation and protective effects on endothelial function between freeze- or spray-dried plasma and its source FFP [110,124], and this may be worth further investigation.

The clinical significance of the changes in each clotting factor is unclear. Fibrinogen and FV are clotting factors that are often depleted in trauma patients with coagulopathy [157,158]. Therefore, the preservation of their function and stability may be the most important factor for reconstituted FDP used for trauma resuscitation. On the other hand, traumatic coagulopathy may be influenced by several interactions between plasma proteins and the surrounding tissues, without clear dominance of single factor [159]. Moreover, the relatively wide variation in factor levels among healthy blood donors means that some thawed or liquid plasma may have lower factor levels than some FDP [94], especially if the FDP is from a single donor (LyoPlas), as opposed to multiple donors (FLyP).

As this review is focused on in vitro studies, the reported studies were mostly conducted in vitro, with a few in vivo and RCTs. Further literature review is necessary to extrapolate our findings and to better understand the clinical outcomes in trauma patients.

RBC, plasma, and platelets are the three main blood components transfused to severely bleeding patients. Plasma is the only one that can be freeze-dried as FDP, with a shelf life of more than one year at room temperature. FDP can be reconstituted within minutes for safe and effective transfusion, providing the aforementioned logistical advantages for administration in a combat and prehospital setting. RBC and platelets have a shelf life of 42 and 5 days when stored at 4 °C and room temperature, respectively, as per current standards of storage [160]. The limited shelf-life stability results in logistical challenges and product wastage.

Prothrombin complex concentrate (PCC) is a lyophilized coagulation factor (factors II, VII, IX, and X) product derived from large donor–pooled plasma and offers a potential alternative to plasma in the management of bleeding [161,162]. The advantages of PCC over FFP include the following: there is no need for ABO blood group matching, lower risk of transfusion-related complications, easier storage and timely administration, and more targeted treatment for factor deficiencies (e.g., elective prophylaxis in patients taking warfarin). However, PCC does not contain the full, balanced complement of procoagulants and anticoagulants that is present in plasma. Therefore, they may be less effective in restoring hemostasis and protecting vascular endothelia and may carry a higher thrombosis risk.

## 6. Future Directions

Large casualties, as seen in the current Ukraine war [163], and as expected in future conflicts when air superiority will not be assured, or during operations in remote, extreme, and austere environments [164], will prohibit immediate evacuation. Therefore, there is a need for blood products, like dried plasma, that can be easily scaled on demand, and exhibit long-term storage with limited-to-no cold chain requirements [165].

Concentrated, low-volume reconstitution of both freeze- and spray-dried plasma has been demonstrated to be safe and effective in the resuscitation of a swine polytrauma model of hemorrhagic shock [128,166]. This could further enhance the logistical advantage (lower volume and weight) in the packaging and transport of dried plasma on the battlefield, as well as in austere environments. The effects of infusing hypertonic, hyper-oncotic fluid are unknown and deserve further evaluation in humans. Future studies are necessary to establish the optimum reconstitution solution and fluid volume, potential immunogenicity changes to dried plasma, and isohemagglutinin influence.

Further modeling and early identification of patients who are most likely to benefit from dried plasma are needed. Additionally, identifying who will be trained to administer it, along with related logistical considerations, will help to clarify future demand for dried plasma in conflicts [30]. Specifically, future research should focus on comparing the efficacy of equal units of dried plasma versus the standard of care with FFP, and different types of dried plasma (licensed and yet to be developed). Dried plasma may be more beneficial in hemorrhagic shock; therefore, categorizing patients by severity may also provide further insight. An in vitro and in vivo comparison between FDP and SDP produced from the same source of human plasma, as opposed to swine plasma [128], would provide a better evaluation of the development process of the two types of dried plasma.

Furthermore, clinical evidence on the use of other concentrates of clotting factors, such as fibrinogen concentrate and PCC, should be compared with dried plasma [162]. These studies should assess meaningful clinical outcomes addressing efficacy within 6 h of injury, as well as mortality, transfusion of blood products, and improvement of coagulopathy. If TXA is administered, it should be documented in both prehospital and hospital settings [85]. Clinical outcomes should include all-cause mortality, organ failure, days in critical care, type and amount of each blood component transfused, physico-chemical parameters (such as body temperature, lactate, base excess, and pH), coagulation/fibrinolysis parameters (such as coagulation factor activities and viscoelastic measurements), and inflammatory parameters [85].

Future studies should also consider the impact of dried plasma on resource allocation (e.g., economic costs, blood product utilization, hospital length of stay). Furthermore, evaluation of use in the prehospital setting should be studied, including challenges to implementation, use, and time to administration.

In addition to coagulation and fibrinolysis factors, the drying effect on microparticle content has not been well reported. One study showed no significant effects of spray-drying on microparticle concentrations in S/D-treated plasma [62]. Microparticles are circulating, phospholipid rich, submicron particles released from the membranes of platelets, blood and endothelial cells [167]. They possess a wide range of biological activities in normal and disease states, including coagulation, thrombosis, and inflammation [168,169,170]. Their abundance and nature in plasma are affected by manufacturing methods such as WB leukoreduction [171] and S/D treatment [62]. The role of microparticles in dried plasma for transfusion efficacy and safety, including trauma resuscitation, deserves further investigation. The relevance of donor and product variability in residual cell counts, microparticle counts, or coagulation parameters for clinical efficacy and safety is still unknown and requires further analysis.

Plasma also consists of hundreds of proteins with therapeutic benefits (e.g., inflammatory mediators) and biological effects unrelated to maintaining hemostatic balance, correction of coagulopathy, including endotheliopathy of trauma, barrier integrity compromise, deregulated coagulation, and overt inflammation in hemorrhaging patients [124,172]. Their biological functions for supporting healthy organs in dried plasma also deserve future investigation. Our previous studies have shown equivalent levels of immuno-inflammatory mediators, such as cytokines, chemokines, and vascular molecules [58], as well as anti-severe acute respiratory syndrome coronavirus 2 (SARS-CoV-2) IgG and neutralizing activity between freeze-dried and source plasma [59].

While dried plasma is an old product, its future is exciting and will likely see a new beginning. The development of dried plasma is on the horizon, as dried plasma is needed for the management of traumatic hemorrhage. Ultimately, blood-derived or synthetic products other than dried plasma will also be needed to fulfill oxygen-carrying capacity and optimize transfusion in remote settings.

## 7. Conclusions

FDP has a long history of use for transfusion in major trauma. At the time of this review, we conclude that there is limited high-level evidence on the therapeutic benefit of current FDP products in bleeding patients. The logistical advantages for military and prehospital settings are evident for the delivery of dried plasma that does not require refrigeration, is temperature stable, light-weight, safe, and can be carried easily, allowing for earlier plasma administration starting at the prehospital setting and continuing into the hospital setting, thereby replacing the use of crystalloid and colloid, which would improve patient outcomes. The logistical and strategic advantages of dried plasma would be even more beneficial for future multi-domain military operations with prolonged evacuation time and interruptions of the supply chain due to mounting geopolitical risks. The new generation of dried plasma would advance the capabilities for prehospital care in far-forward areas from the military, civilian, and disaster-preparedness perspectives. Low-volume reconstitution is effective, whereas the optimal fluid for reconstitution continues to be investigated. Logistical advantages for implementation in austere environments and existing unmet clinical needs should be carefully considered for the further development of next-generation dried plasma through laboratory and clinical research.

## Figures and Tables

**Table 2:** Comparison of hemostatic properties among different freeze- and spray-dried plasma products. The values in bold are out of clinical reference range per test instrument. Where units were originally reported as units/mL (U/mL) or international units/mL (IU/mL), they have been converted to percent normal (%), where 100% is equal to 1.00 U/mL or IU/mL as appropriate. Unless specified, data represent mean ± standard deviation.

Product Name	FLyP (% Changes Relative to Source Plasma)	LyoPlas (% Changes Relative to Source Plasma)	Normal Range/ Physiological Norms/Reference Range/Clinical Reference	Teleflex FDP (% Changes Relative to Source Plasma)	CFDP (% Changes Relative to Source Plasma)	TFDP (% Changes Relative to Source Plasma)	OctaplasLG Lyo (% Changes Relative to Source Plasma)	Spray-Dried Plasma (ODP) (% Changes Relative to Source Plasma)
INR		1.05 ± 0.12	0.8–1.2///0.9–1.1	**1.11** ± 0.1 ^a^/1.1 ± 0.1 ^b^ (7.06/8.33%)				
PT (s)	/1.2 ± 0.1 ^c^ (8%)	Not reported	9.4–12.5	**12.9** ± 1.0 ^a^/**12.6** ± 0.9 ^b^ (7.19/8.52%)	14.6 ± 0.6/13.6 ± 0.3 (7.35/5.9%)	11.3 ± 0.67 (4.1%)	11.4 (−3.39%)	12.5 ± 0.9 (10%)
PTT (s)	41.0–41.5/39.0 ± 2.4 (/11%)	37.9 ± 4.3/32.22 ± 3.46 (12.8%)	26–37/30–40/30–40/25.1–36.5	31.1 ± 2.1/33.2 ± 2.7 (5.15/6.41%)	40.7 ± 3.3/36 ± 2 (8.53/6.9%)	29.4 ± 2.5 (4.9%)	29 (3.57%)	29.9 ± 3.4 (2%)
Fibrinogen (g/L)	2.4 ± 0.3 (−11.1%/0%)	2.6 ± 0.3/3.256 ± 0.139 (−3.7%)	2–4/2–4/2–4/2–3.93	2.372 ± 0.408/ 2.509 ± 0.557 (−6.83/−4.81%)	2.54 ± 0.23/2.9 ± 0.1 (−3.42/−3.0%)	3.02 ± 0.58 (0.5%)	3.1 (3.33%)	2.14 ± 0.39 (−18%)
Pro-thrombin (%)								86 ± 11 (−11%)
FII (%)			///79%–131%	82.2 ± 9.6/**77.7 ± 7.9** (−11.87/−10.19%)	/78 ± 4 (−4.9%)		115 (6.48%)	
FV (%)	70/**0.51 ± 16** (−30%/−25%)	98.9 ± 7.5/107.38 ± 8.2 (−9.1%)	65–150/0.7–1.2/70–120/62–139	73.8 ± 13.7/79 ± 12.9 (−10.05/−14.67%)	61 ± 7/83 ± 8 (−20.78/−9.8%)	81 ± 14 (−3.5%)	90 (−3.23%)	104.4 ± 20.2 (6%)
FVII (%)			///50–129	75.4 ± 18.5/77.1 ± 18.0 (−7.15/−10.12%)	/92 ± 4 (/−7.1%)		113 (5.61%)	92.3 ± 20.2 (−9%)
FVIII (%)	70/62 ± 10 (−12.5%/−20%)	80 ± 20/79.12 ± 12.44 (−21.6%)	60–150/0.5–1.5/0.5–1.5/50–150	129.8 ± 28.9/107.9 ± 29.1 (−10.11%/−6.85%)	52 ± 14/60 ± 20 (/−14.3%)	75 ± 23.5 (−12.8%)	93 (−13.89%)	106.5 ± 27.2 (−17%)
FIX (%)			65–150	92.8 ± 13.4/95.3 ± 17.2 (−13.08%/−13.50%)	/73 ± 5 (−5.2%)		120 (4.35%)	109.4 ± 17.0 (−9%)
FX (%)			77–131	73.4 ± 11.7/81.2 ± 13.2 (−11.65%/−14.35%)			125 (14.68%)	88.9 ± 14.6 (−12%)
FXI (%)	70/79 ± 11 (16.7%/6%)	81.7 ± 6.6/98.48 ± 12.47 (−10.5%)	65–150/0.5–1.4/50–140/65–150	97.9 ± 24.0/102.9 ± 17.8 (−10.94%/−4.63%)	/100 ± 8 (−2.2%)		80 (−13.98%)	105.4 ± 19.8 (−5%)
FXII (%)			50–150	80.9 ± 17.0/83.1 ± 21.2 (−11.37%/−15.73%)			100 (−15.97%)	88.8 ± 21.4 (−15%)
FXIII (%)	/103 ± 12 (/3%)		//20–120		/110 ± 2 (−3.0%)		90 (−2.17%)	98.2 ± 18.3 (−26%)
Antiplasmin (%)	90/95 ± 30 (12.5%/1%)	82.4 ± 4.6	/80–120/80–120/98–122	**86.9** ± 9.9/**92.5** ± 6.8 (−6.53%/−9.28%)	/60 ± 10 (−14.3%)		47 (9.30%)	99 ± 8 (−1%)
Antithrombin (%)	1.0/1.01 ± 0.05 (0%/−3%)	0.91 ± 0.05/92.2 ± 6.37% (−9%)	80–135/0.8–1.2/80–120/83%–128%	0.850 ± 0.096/0.907 ± 0.098 (−5.49%/−7.07%)	0.90 ± 0.04/0.84 ± 0.05 (−6.25/−8.7%)		1.06 (10.42%)	0.91± 0.13 (−1%)
D-dimer (%)					30 ± 7/29 ± 8 (0/−3.3%)		10.8 (−12.90%)	46 ± 23 (−10%)
Plasminogen (%)		91.1 ± 2.9 (−11.2%)	///80.2–132.5	82.3 ± 13.5/86.8 ± 17.4 (−4.92%/−8.49%)	100 ± 3 (−4%)		86 (−1.15%)	103 ± 13 (3%)
Protein C (%)	90/96 ± 9 (0%/0%)	/100.4 ± 14.23	80–150/0.7–1.2/70–120/70–140	89.2 ± 15.5/90.5 ± 15.6 (−6.33%/−9.28%)	86 ± 10/91 ± 7 (/−1.6%)	108 ± 19.7 (1.9%)	103 (3%)	98 ± 20 (−7%)
Protein S activity (%)	90/77 ± 16 (−11.1%/−7%)	78.3 ± 7.2 (2.2%)	60–150/0.7–1.4/70–140/54.7–146.1	90.8 ± 15.4/92.7 ± 13.5 (−1.17%/−7.48%)	69 ± 5/79 ± 7 (−6.76/−12.1%)	96 ± 14.1 (3.2%)	67 (1.52%)	98.0 ± 19.2 (−8%)
Free Protein S (%)		/92.36 ± 19.67					81 (−6.90%)	96.4 ± 20.5 (−7%)
vWF/RiCoF (%)		60.7 ± 18.1 (−25.0%)					95 (9.20%)	62.3 ± 23.5 (−40%)
vWF activity (%)			///40.3–163.4	121.4 ± 35.5/111.4 ± 40.0 (−7.13%/−10.52%)				
vWF antigen (%)		/76.72 ± 38.3	60–160//42.0–176.3	142.8 ± 40.1/123.0 ± 44.0 (−9.87%/−9.13%)	/100 ± 40 (/−9.1%)			142 ± 42 (6%)
ADAMTS13 (%)					/120 ± 10 (/−14.3%)		92 (−7.07%)	135.0 ± 5.3 (5%)
References	Sailliol et al., 2013 [48]/Martinaud et al., 2012 [69]	Bux et al., 2013 [49]/Zur et al., 2019 [113]	Zur et al., 2019 [113]/Sailliol et al., 2013 [48]/Martinaud et al., 2012 [69]/Cancelas et al., 2022 [93]	Cancelas et al., 2022 [93]	Peng et al., 2022 [59]/Sheffield et al., 2022 [94]	Flaumenhaft et al., 2021 [91]	Heger and Gruber 2022 [92]	Liu et al., 2019 [98]

^a^ Produced from anticoagulant citrate dextrose FFP; ^b^ citrate phosphate dextrose FFP; ^c^ prothrombin time (ratio test/control). ADAMTS-13, a disintegrin and metalloproteinase with a thrombospondin type 1 motif, member 13; aPTT, activated partial thromboplastin time; PT, prothrombin time; vWF/RiCoF, von Willebrand factor/ristocetin cofactor.

**Table 3:** Comparison between spray-dried and freeze-dried plasma products.

Parameter	Spray-Dried Plasma	Freeze-Dried Plasma
Highest retention of clotting factors in reference to fibrinogen, FV and FVIII	−18%, +6%, −17%	0%, −3%, −7%
Particle size	Powder from droplets	Bulk material (no droplets)
Rate of formation	Few hours or less	One to several days
Reconstitution time	One to few minutes	Few minutes
Plasma source	Intended for single donor	Single or pooled donors
Autologous plasma	Easy to apply	Possible
Throughput	Low to medium	Medium to high
Equipment	Desk top	Mainframe
Cost	Cheaper, more energy efficient	Two to three times more than frozen plasma
Production sites	Possibly many, distributed	Often few, centralized
Current status	Preclinical development and phase I clinical trial	Well-studied clinical safety and efficacy

## Data Availability

The raw data supporting the conclusions of this article will be made available by the authors on request.
